# Potential for Early Noninvasive COVID-19 Detection Using Electronic-Nose Technologies and Disease-Specific VOC Metabolic Biomarkers

**DOI:** 10.3390/s23062887

**Published:** 2023-03-07

**Authors:** Alphus Dan Wilson, Lisa Beth Forse

**Affiliations:** 1 Pathology Department, Center for Forest Health & Disturbance, Forest Genetics and Ecosystems Biology, Southern Research Station, USDA Forest Service, Stoneville, MS 38776, USA; 2 Southern Hardwoods Laboratory, Southern Research Station, USDA Forest Service, Stoneville, MS 38776, USA

**Keywords:** COVID-19, early disease diagnosis, e-nose devices, disease-specific biomarkers, metabolomic biomarkers, metabolite profiles, pathophysiology, point-of-care testing, volatile organic compounds (VOCs)

## Abstract

The established efficacy of electronic volatile organic compound (VOC) detection technologies as diagnostic tools for noninvasive early detection of COVID-19 and related coronaviruses has been demonstrated from multiple studies using a variety of experimental and commercial electronic devices capable of detecting precise mixtures of VOC emissions in human breath. The activities of numerous global research teams, developing novel electronic-nose (e-nose) devices and diagnostic methods, have generated empirical laboratory and clinical trial test results based on the detection of different types of host VOC-biomarker metabolites from specific chemical classes. COVID-19-specific volatile biomarkers are derived from disease-induced changes in host metabolic pathways by SARS-CoV-2 viral pathogenesis. The unique mechanisms proposed from recent researchers to explain how COVID-19 causes damage to multiple organ systems throughout the body are associated with unique symptom combinations, cytokine storms and physiological cascades that disrupt normal biochemical processes through gene dysregulation to generate disease-specific VOC metabolites targeted for e-nose detection. This paper reviewed recent methods and applications of e-nose and related VOC-detection devices for early, noninvasive diagnosis of SARS-CoV-2 infections. In addition, metabolomic (quantitative) COVID-19 disease-specific chemical biomarkers, consisting of host-derived VOCs identified from exhaled breath of patients, were summarized as possible sources of volatile metabolic biomarkers useful for confirming and supporting e-nose diagnoses.

## 1. Introduction

The high human and economic costs of the COVID-19 worldwide pandemic, caused by the severe acute respiratory syndrome coronavirus (SARS-CoV-2), have challenged healthcare providers with the difficult tasks of detecting the viral pathogen in both symptomless carriers and symptomatic patients as well as developing effective therapeutic treatments. Current methods developed for this task in large-scale, mass-screening efforts initially utilized semi-invasive sampling methods involving somewhat painful deep nasal swabs of sinus tissue to acquire clinical samples for diagnostic testing. The discomfort associated with COVID-19 sampling coupled with low detection accuracy for early COVID-19 infections, persistent positive results after infection, the delay in receiving results, and fears of being quarantined or isolated have discouraged many from getting tested and reduced the utility of diagnostic results [1,2]. These problems have pointed to the need for new noninvasive detection methods that provide effective and efficient results in point-of-care testing (POCT) situations where rapid, high-volume, accurate testing results are required. Numerous other problems associated with conventional COVID-19 detection methods have prompted the continuous development of improvements in COVID-19 sampling and detection methods [3].

Four primary methods have been developed for SARS-CoV-2 detection, including nucleic acid (NA)-based testing, antibody-based testing, antigen based-testing, and computed tomography (CT) chest scans [4]. The use of CT chest scans produces results with high accuracy and sensitivity (67–100%), but low specificity (25–80%), and they do not distinguish between pneumonia caused by SARS-CoV-2 from other types of viral pneumonia [5]. The quantitative real-time reverse transcription polymerase chain reaction (rRT-qPCR) method is still the gold standard for NA-testing of SARS-CoV-2 infections because it is very reliable and offers high sensitivity and specificity [5,6,7,8,9]. Nevertheless, most current PCR-based methods do not currently support rapid and point-of-care diagnosis, although recent studies have reported development of ultrafast PCR systems for COVID-19 detection [10,11,12]. You et al. **[10]** performed NA amplification in a nano-localized environment to significantly increase the thermocycling rate, but this improvement has not been widely implemented. Other COVID-19 detection methods based on antibodies, cytokines, RNA, and portable biosensors were discussed by Taleghani and Taghipour [13].

Current PCR-based technologies generally provide slow delivery of results 1 to 2 days after sampling which allow further SARS-CoV-2 transmissions to occur before symptoms appear [14]. Asymptomatic individuals may have similar viral loads to those with symptoms. Consequently, more rapid antigenic tests such as enzyme-linked immunosorbent assays (ELISA), providing results within 10−30 min and sensitivity up to 90%, are routinely used as prescreening methods. These tests also have limitations in that they require properly equipped testing sites and trained personnel which may cause logistic challenges associated with limitations of test product supply chains due to the large number of tests that must be performed each day for large populations. In addition, rapid tests similarly require somewhat painful or unpleasant semi-invasive swab sampling of nasopharyngeal and oropharyngeal cavities with the aim of acquiring sufficiently high viral test loads, although this goal is not always met, sometimes leading to false-negative test results. These problems with various detection methods have delayed acquisition of COVID-19 test results, sometimes precluding more effective early treatments [15].

Recent developments of electronic-nose (e-nose) and related gas sensing technologies have demonstrated the potential to help slow the spread of contagious diseases through rapid early detection of diagnostic volatile organic compounds (VOCs), including disease-specific biomarkers present in the exhaled breath of infected patients, particularly prior to symptom development [16,17]. Understanding the biochemistry and metabolic effects of COVID-19 are critical to revealing specific target VOC gases, emitted in the breath of severe acute respiratory syndrome coronavirus (SARS-CoV-2) infected individuals, necessary for effective sensor array selection for e-nose disease detection and VOC quantification using selective breathomics-based diagnostic screening tools [18,19,20]. Thorough studies on the metabolomics and pathogenesis of COVID-19 are needed to elucidate the mechanisms by which the viral pathogen affects and alters the metabolic pathways of a human host to generate specific VOCs in certain organ systems [3,20,21,22,23].

Electronic-nose instruments have been developed for numerous healthcare applications ranging from detection of infectious diseases caused by numerous microbial pathogens [24,25,26], to prescreening detection of genetic disorders [27,28], and noninfectious diseases (such as asthma and COPD [29,30,31], cancer [32,33,34,35], cardiovascular diseases [36,37,38], diabetes [39], predicting effectiveness of chemotherapy treatments [40], and mental health [41] to long-term disease monitoring [17,42]. These devices represent various types of electronic aroma detection (EAD) technologies capable of discriminating between a wide range of complex gas mixtures composed of relatively low-molecular-weight (<300 Daltons) biomarker metabolites, primarily volatile organic compounds (VOCs) derived from a wide diversity of chemical classes [43]. E-nose devices with multisensor arrays discriminate between complex VOC mixtures, released in the headspace of clinical air samples, through the production of unique sensor–response patterns (smellprint signatures) specific to diseases and use of specialized statistical models and pattern recognition algorithms to facilitate sample discriminations [44,45].

Previous reviews on applications of e-nose technologies using breath-based analyses for COVID-19 disease detection are limited and none have addressed the full range of electronic and e-nose devices tested or the important COVID-19-specific metabolomic VOC biomarkers that have been identified [15]. Miller et al. [3] mentioned clinical detection of COVID-19 in patients’ breath using electronic-nose devices and discussed the need for future work to further develop e-nose devices for detecting COVID-19 complex mixtures of VOC inflammatory biomarkers. In addition, they focused on neurological complications of COVID-19 associated with viral infection routes from olfactory epithelium in the nasal cavity to the central nervous system (CNS) and its angiotensin-converting enzyme 2 (ACE2) interaction. Subali et al. [46] examined six studies comparing the sensitivity and specificity of VOC-based e-nose breath analysis diagnostic performance for SARS-CoV-2 infection compared with RT-PCR. Their subgroup analysis using gas chromatography-mass spectrometry (GC-MS) and pattern recognition with e-nose devices revealed higher sensitivity in the e-nose group and more promising specificity for COVID-19 public screening. The current review provides a more detailed and comprehensive evaluation of e-nose and breathalyzer-type devices that have been developed for early COVID-19 detection.

This review summarizes considerable evidence showing the strong potential for using electronic-nose and related VOC sensor devices to noninvasively detect and monitor COVID-19 human infections at early and later stages of disease development in asymptomatic, presymptomatic, and symptomatic individuals. We include many identified COVID-19-associated exhaled breath VOCs from different chemical classes now known as potential targets for e-nose detection which were identified in clinical samples. These COVID-19 VOC biomarkers may be noninvasively detected collectively as complex mixtures using e-nose devices without discomfort or anxiety to patients. Furthermore, we summarize and compare major world viral pandemics and their associated differences in physical manifestations on human populations compared with COVID-19-specific symptoms. We describe how these differences in COVID-19 symptomology result from different mechanisms of disease, affecting different metabolic pathways in multiple organ systems, generating unique organ- and pathway-specific VOCs. We describe and evaluate current standard diagnostic testing methods used for COVID-19 detection and expounded on experimental and clinical results of e-nose detection methods being developed for cheaper and more rapid COVID-19 detection. Individual e-nose devices were evaluated for accuracy with associated advantages and disadvantages for use in different application settings, including clinical and POCT sites where COVID-19 disease diagnostics are performed.

## 2. Factors Affecting E-Nose Early Detection of Human Disease

Investigations into the potential for using e-nose devices for early COVID-19 disease detection necessarily require considerations of various extraneous or intervening factors that may affect or possibly interfere with disease diagnoses based on VOC emissions. A thorough assessment relative to the analysis of VOC-emissions from clinical samples has indicated that most extraneous and intervening factors fall into categories, including the state of a patient’s health, existence of other coinfections of the lungs or upper respiratory tract, their history of pre-existing conditions, exposure to exogenous gases, diet, predisposing risk factors, age classes, race, responses of affected organs, physiological pathways affected within individual organs, and volatility of produced disease-associated metabolites. Schematic diagrams showing some pertinent factors causing variations in VOC within human exhaled breath were provided by Phillips et al. [47].

Human breath composition may change considerably in association with numerous variables that must be taken into consideration when sampling of exhaled breath for the purposes of e-nose COVID-19 diagnostics and VOC breathomic studies. There are a number of procedural clinical practices used to minimize the temporal variability of breath composition to provide more consistent data in assessing SARS-CoV-2 infections. Patients are often required to abstain from eating any foods, smoking tobacco, vaping nicotine, taking oral drugs, or drinking anything but water for a minimum of 3–7 h prior to breath sampling. Oral cavities are often rinsed and the patient may be required to breathe purified and carbon-filtered air, devoid of VOCs, for a period prior to breath air sampling. Other strategies to avoid the adverse affects of variable factors on breath VOC emissions include the use of creative statistical procedures. Individuals with a habit of alcohol, tobacco, or drug use may be placed into separate sampling categories for more effective statistical comparisons. For example, confounding dietary effects on breath VOC emissions monitoring may be processed out during statistical modelling by adjusting sampling schedules around mealtimes to prevent the introduction of dietary artefacts into data analyses [48]. Common exogenous VOCs in the human breath may be corrected by subtracting the background VOCs in room air from the VOCs observed in the breath [47].

### 2.1. States of Human Health

Electronic-nose devices are well known for the versatile capabilities of measuring and monitoring the overall health condition of clinical patients having many variable states of health [29,36,45]. The acquisition of VOC patterns from patients, recorded as an e-nose smellprint signature, provides a snap-shot indication of a patient’s health state, based on breath VOC profiles, at a single point in time. Multiple periodic acquisitions of e-nose smellprint data over time allow for the monitoring of a patient’s health state continuously to determine the rate of recovery from disease and provide improved indications of more accurate prognoses as more data is acquired [40,42]. The normal VOC profiles of relatively healthy patients can vary considerably with age, dietary habits, level of nutrition, drug use, exposure to environmental pollutants, level of exercise, strength of immune system responses, and many other factors. These multiple variables affecting a patient’s VOC profile must be considered while assessing health states based on VOC emissions.

The most effective approach for assessing a patient’s health state is to acquire a history of personal VOC profiles over time during known periods of relative good health and during episodes of known sicknesses diagnosed by conventional methods recommended for individual diseases. A historical record of VOC profiles of individual patients under various states of health can provide valuable reference data indicating how an individual’s unique physiology and immune system responses to specific diseases are reflected in their corresponding real-time VOC profiles. In this way, e-nose devices may be used as effective tools to monitor changes in VOC profiles of individual patients in various states of health. Likewise, similar data of VOC profiles obtained from many healthy and COVID-19 patients, with and without disease symptoms, may be used to investigate and determine any commonality of metabolic effects induced by the disease which may be reflected by changes in VOC profiles in the majority of patients infected by the SARS-CoV-2 coronavirus.

Further metabolomic investigations could identify the presence of unique VOC metabolites, consistently associated with COVID-19 pathogenesis, which might serve as effective early chemical biomarkers of the disease prior to symptom development. Disease biomarkers provide an additional source of confirmation of diagnoses determined initially from unique e-nose smellprint signatures indicative of specific diseases [49]. The detection of multiple specific disease VOC biomarkers strengthens this secondary confirmation particularly for biomarker metabolites that are not known to be produced in association with any other known disease or in healthy COVID-19-negative individuals.

### 2.2. Location and Occurrence of COVID-19 Disease Effects

The location of disease within different compartments and organs of the body can determine the types of host metabolic pathways affected by COVID-19 disease processes. For example, variations in biochemical processes associated with specialized functions of differentiated tissues within different organs and organ systems influence the interactions and responses of host tissues to host–pathogen metabolic interactions. Furthermore, the types of host metabolic changes (abnormal diversions in metabolic pathways) induced by pathogenesis are strongly influenced by the types and categories of microbial pathogens attacking healthy tissues [18]. This phenonomenon is easily explained by the many unique molecular mechanisms (e.g., pathogenic determinants) by which different types of pathogens initiate and generate diseased states within the body. This information is often determined by metabolomic studies investigating the precise groups of host metabolic pathways affected by specific disease-causing agents. Consequently, abnormal metabolic pathways (generated by pathogenesis) often lead to the production of unique volatile metabolites that may be identified and associated with specific pathogens and associated diseases [18]. In many cases, specific VOC-metabolites may be derived from both the altered host metabolisms (pathways affected) or from the metabolism of the pathogen itself. These unique pathogen- and disease-associated metabolites often serve as effective chemical biomarkers of specific diseases, particularly useful as targets for e-nose disease detection. In the case of noncellular viral pathogens, most VOC disease biomarkers are derived primarily from abnormal alternations in host metabolic processes induced by pathogenesis.

The unique characteristics and differences in viral disease mechanisms associated with different types of coronavirus infections of human and nonhuman host cells result in unique alterations in physiological processes with associated distinct manifested symptoms, depending on the combination of affected organs and metabolic pathways. These differences in the effects of major viral pathogens on human organ systems with associated symptomology are summarized for the most important viral diseases responsible for global pandemics in recent human history in Table 1. A list of abbreviations for respiratory-related human viral diseases causing global pandemics, analytical VOC-sensitive instruments used for early disease detection, instrument sensor types and arrays, clinical methods, and VOC-metabolite (analyte) categories cited in this review is provided just before the References section.

The major viral pandemics that have occurred in the 20th and 21st century are taxonomically divided into three major viral families: the Orthomyxoviridae, Retroviridae, and Coronaviridae. The Orthomyxoviridae consist of negative-sense single-stranded RNA viruses that are responsible for most of the historical flu pandemics. The Retroviridae and Coronaviridae families contain both positive-sense and single-stranded RNA viruses and are the etiologic agents that have caused HIV, SARS, and Camel flu pandemics, as well as the current COVID-19 pandemic. Differences in the genetic construct of viral genomes influence the mechanisms by which viruses from individual families attack their host and initiate disease. These differences result in widely varying combinations of manifested symptom types due to variable effects of viral replication and controls on host gene dysregulation initiated within individual infected organs and tissues of human hosts.

The disease mechanisms by which different viral groups take control of human cells and initiate replication cycles are determined to a significant extent by the relative susceptibility and level of involvement in which individual organ systems are attacked, the associated metabolic pathways affected, and the types and severity of damage sustained over short- and long-term time scales. Some organ systems sustain short-term damage that can be repaired to restore normal functions. However, other organs may become dysfunctional due to chronic persistent viral attacks with prolonged disease mechanisms that may result in lasting effects including permanent organ damage with reduced or limited capabilities for restored functions. For example, the susceptibility of individual patients to both acute COVID-19 and long-COVID-19 effects can vary widely and depend on a complex combination of many predisposing factors including race, obesity, general level of health and immune response, nutritional state, prior exposures to SARS-CoV-2 at various levels, and many genetic factors affecting unique metabolic processes occurring within specific organ systems of different individuals [49,65,66].

Disease mechanisms associated with different pandemic viruses result in unique combinations of symptoms and physical effects in different individuals. The types and range of manifested symptoms of various viral infections can change dramatically during the disease process until the individual eventually recovers. As a virus mutates following multiple passings and disease cycles through susceptible hosts, the disease mechanism can change, as new strains form, that may result in different intensities and symptomology in future infected individuals and new or different organ systems may be affected. The SARS-CoV-2 coronavirus has caused combinations of symptoms that have differed significantly from those of previous viral pandemics. Differences in viral biochemical effects ultimately result in changes in human host metabolic pathways in various ways that determine the types and quantitative effects of viral infection on VOC emission within the breath of SARS-CoV-2-infected patients which may be detected by e-nose and advanced electronic chemical-analysis technologies.

### 2.3. Pathophysiological Effects of SARS-CoV-2 Coronavirus on Human Hosts

Viral pathogens take control of cellular machinery and initiate viral replication mechanisms that disrupt a number of important host metabolic pathways. Viral pathogenesis may lead to significant effects on human organ systems and generate disruptions in critical physiological and neurological functions, potentially leading to long-term physical damage, lasting adverse symptoms, and occasionally death. The following discussion provides an overview of the wide range of COVID-19 symptom groupings and associated biochemical and physiological effects of SARS-CoV-2 infections on different age groups, races, and patient classes that ultimately result in detectable changes in exhaled breath VOCs relative to healthy individuals.

#### 2.3.1. Multisystem Inflammatory Syndrome in Children

The majority of children and early adolescents acquiring SARS-CoV-2 infections are asymptomatic or have only mild to moderate illness, but severe or critical illnesses do occur in rare cases [49,67,68]. Examples of differential lingering effects and damage caused by COVID-19 in individual patients with various predisposing factors are largely dependent on the combination and extensiveness of organ systems affected and the resulting diversity of associated symptoms manifested, which are all expressed among cases of Multisystem Inflammatory Syndrome in children (MIS-C). The first reported occurrence of MIS-C, observed initially only in children (ages 4–15) with hyperinflammatory shock symptoms similar to Kawasaki disease and toxic shock syndrome, occurred in England (April 2020) during the peak of the COVID-19 pandemic in Europe [69]. A unique combination of symptoms, presumed to have occurred 2–4 weeks post-acute COVID-19 based on serological evidence of infection with SARS-CoV-2, were discovered to be divided into three distinct clusters (symptom categories) that were strongly associated with viral attack and damage to specific organs systems [65]. A total of 570 MIS-C patients who met the case definition were almost exclusively SARS-CoV-2-positive, determined by reverse transcription–polymerase chain reation (RT-PCR), and characterized by elevated inflammation markers, abdominal pain, cardiac dysfunction, and shock. Of these 570 total patients, 63.9% required treatment in an intensive care unit (ICU), and 10 patients (1.8%) died. MIS-C generally caused adverse effects involving at least four organ systems, primarily and commonly including gastrointestinal (90.9%), cardiovascular (86.5%), dermatologic or mucocutaneous (70.9%), and hematologic (73.9%) involvement. The majority of patients (67%) did not have preexisting underlying medical conditions prior to MIS-C onset [69].

Individuals in the first patient class exhibited additional clinical signs and symptoms of conjuctivitis, peripheral edema, rash, fever, gastrointestinal symptoms, and elevated markers for cardiac damage. Class 1 patients (35.6%) had the highest number of organ systems affected (six or more involved) with cardiovascular and gastrointestinal being the most commonly affected [65]. The patients in this group had significantly higher prevalences of myocarditis, abdominal pain, shock, lymphopenia, elevated C-reactive protein (indicative of liver inflammation), ferritin (accute-phase reactant), troponin (indicative of cardiac damage), brain natriuretic peptide (BNP), or proBN (indicative of heart failure). By contrast, class 2 patients (29.6%) had somewhat reduced cardiovascular and gastrointestinal involvement and the lowest incidence of dermatologic and mucocutaneous involvement, but significant respiratory system involvement, and were more likely to have shortness of breath, coughing, pneumonia, and acute respiratory distress syndrome (ARDS), suggesting that their illness primarily developed from acute COVID-19. These patients exhibited the highest fatality rate among the three classes (5.3%). Class 3 patients had the lowest prevalence of organ system involvement (four or less) and the lowest incidence of underlying medical conditions, complications (including shock and myocarditis), and markers of cardiac damage and inflammation. The hematological organ system was least prevalent in class 3 patients among the three classes. Class 3 patients also more commonly met the criteria for Kawasaki disease (6.6%), compared with class 1 (4.9%), and class 2 (3.0%). Patients in class 3 were further distinguished by the highest incidence of rash (62.6%) and mucocutaneous lesions (44.9), and a higher prevalence of coronary artery aneurysm and dilatations than class 2 patients, but lower than in class 1 patients. The neurologic and renal organ systems, as well as others such as periorbital (4.7%) and cervical (13.3%) organ systems were least affected in MIS-C patients of all three classes.

The most commonly reported underlying medical condition for MIS-C patients was obesity, occurring in 30.5% of Hispanic, 27.5% of Black, and 6.6% of White MIS-C patients. Hispanic and Black patients (combined) accounted for the largest proportion (73.6%) of reported MIS-C patients. Similarly, these two groups have been reported to be disproportionally affected by acute COVID-19 [10], often with higher risk for COVID-19 and more severe illness, possibly including more adverse effects of MIS-C [65].

The adverse affects of COVID-19 on multiple organs were quantitied and categorized based on the percentage of all patients having symptoms associated with specific organs and the relative involvement of each, primarily including seven organs: gastrointestinal (90.9%), cardiovascular (86.5%), hematologic (73.9%), dermatologic (70.9%), respiratory (63.0%), neurologic (38.2%), and renal (18.4%) effects in decreasing order [65]. Gastrointestinal effects were manifested mainly as abdominal pain, vomiting, and diarrhea. Cardiovascular symptoms included congestive heart failure, cardiac dysfunction, coronary artery dilatation or aneurysm, elevated BNP or troponin, hypotension, mitral regurgitation, myocarditis, pericardial effusion, and shock. Dermatologic and mucocutaneous symptoms involved conjunctival injection, mucocutaneous legions, and rash, whereas hematological manifestations were elevated D-dimer, thrombocytopenia, and lymphopenia. Respiratory effects included acute respiratory distress syndrome (ARDS), cough, shortness of breath, chest pain or tightness, pleural effusion, and pneumonia. Headache was the only neurologic symptom evaluated and acute kidney injury was the sole renal symptom recorded. Other minor miscellaneous effects included cervical lymphadenopathy and periorbital edema.

A multicenter study estimated that there was an 80% increase in new-onset type 1 diabetes (T1DM) in children during the COVID-19 pandemic [70]. They found that angiotensin-converting enzyme 2 (ACE2) receptor, the binding site for SARS-CoV-1 and -2, was strongly expressed in pancreatic endocrine cells. They speculated that the SARS-CoV-1 virus may have entered pancreatic islet cells via the ACE2 receptor leading to β-cell damage and new-onset, transient diabetes. They further postulated that SARS-CoV-2 viral exposure contributed to an observed increase in T1DM in children by precipitating or accelerating type 1 diabetes onset.

#### 2.3.2. Multisystem COVID-19 Effects on Adults

The current concensus is that COVID-19 may be considered to be equally categorized as an acute multiorgan disease in adults as it is in severe cases for children [71]. The SARS-CoV-2 coronavirus does not merely cause a localized respiratory infection affecting the lungs, but a systemic, multisystem disease affecting many organs and involving multiple interlinked interactions between complex processes resulting in immunological, inflammatory and coagulative cascades [49]. Major extrapulmonary clinical manifestations (symptoms) in COVID-19 patients include the following (with relative frequency percentages listed in decreasing order), based on 4200 total patients: fatigue (72.22%), ageusia or impaired sense of taste (58.73%), appetite loss (52.78%), anosmia or impaired sense of smell (46.83%), heart palpitation (33.33%), headache (33.17%), nausea/vomiting (31.43%), and diarrhea (25.40%) [72]. The frequencies (%) of symptoms occurring among symptomatic COVID-19 patients include the following (in decreasing order): sinus tachycardia (95.56%), lymphocytopenia (38.49%), hepatitis (36.83%), leukopenia (35.48%), gastroenteritis (27.83%), sepsis (22.22%), proteinuria (20.87%), coronary artery disease (17.30%), and acute kidney injury (16.34%) [72]. The prevalence of coagulation defect-associated disorder (CDAD) was attributed to deep venous thrombosis in 15.56% of patients, acute coronary syndrome in 7.78%, brain infarction in 6.35%, pulmonary artery thrombosis in 3.25%, and SMA thrombosis in 0.32% of symptomatic patients [72].

The most frequently reported early extrapulmonary manifestations include deep venous thrombosis, non-specific abdominal symptoms, hypogeusia, hyposmia, and corneal congestion [73]. Some additional rarer extrapulmonary manifestations in certain organs and locations such as in the brain, eyes, ears, intestines, myocardium, peripheral nerves, muscles (aches), skin, and vessels also have been reported upon onset of COVID-19. Gut microbiome composition is often significantly altered in COVID-19 patients compared with non-COVID-19 individuals irrespective of whether patients had received medication (*p* < 0.01) [74]. Perturbed microbiome composition increases with disease severity along with elevated plasma concentrations of blood markers and inflammatory cytokines, such as C-reactive protein (CRP), aspartate aminotransferase, gamma-glutamyl transferase, and lactate dehydrogenase. These associations between gut microbiota composition, levels of cytokines, and inflammatory markers in COVID-19 patients suggest that the gut microbiome may be affect the magnitude of COVID-19 severity by possibly modulating host immune responses [74]. Several gut commensals including *Faecalibacterium prausnitzii*, *Eubacterium rectale*, and bifidobacteria with known immunomodulatory potential were underrepresented in COVID-19 patients and remained low up to 30 days after disease resolution. Gut microbial dysbiosis after disease resolution could contribute to persistent symptoms (long-COVID), highlighting the need to better understand how gut microorganisms may modulate COVID-19-induced inflammation.

Multiple mechanisms proposed to explain the pathobiology of liver damage include ACE2 receptor cholangiocytes-mediated systemic inflammation, cytokine storm, hyperinflammation, and hypoxic changes [75]. Pre-existing liver and GI diseases may increase susceptibility to COVID-19 damage by altering tissue expression and distribution of viral entry via ACE2 receptor along with host protease TMPRSS2, which is also required for both spike protein binding and cleavage to initiate viral infection [75]. Altered immune status due to pre-existing conditions may result in delayed SARS-CoV2 virus clearance or prolonged viremia. Even though GI and hepatic manifestations of are less severe, the detection of SARS-CoV2 virus in a patient’s stool indicates GI tropism, virus replication and shedding from the GI tract. Thus, COVID-19-induced liver injury, acute hepatic decompensation, and incidences of acute-on-chronic liver failure may change the outcomes of liver and GI diseases. COVID-19 patients often have liver injury. High levels of interleukin-6 (IL-6) and its circulating receptor, which form a complex to induce the inflammatory signal, have been observed in patients with COVID-19 [76]. The pathogenesis of liver involvement in COVID-19 includes the secondary effect of immune dysregulation, hypoxia from respiratory failure, ischemic damage caused by vascular endotheliitis, congestive heart failure, drug-induced liver injury, and viral cytotoxicity. Patients with cirrhosis, chronic liver diseases, and hepatocellular carcinoma are at high risk of acute COVID-19 and mortality [77].

The SARS-CoV-2 virus causes a complex pulmonary disease that includes pneumonia with vascular leakage, resulting in respiratory failure, acute respiratory distress syndrome (ARDS), and many other extrapulmonary manifestations of COVID-19 affecting the cardiovascular, dermatologic, endocrinologic, gastrointestinal, hematologic, hepatobiliary, myocardial, neurologic, ophthalmologic, pharyngeal, and renal systems in variable combinations in different individuals, each with unique physiochemical susceptibilities, immunities, and health states [78,79,80]. Multi-organ injury secondary to SARS-CoV-2 infection results in endothelial cell damage, thromboinflammation, immune response dysregulation, and dysregulation of the renin–angiotensin–aldosterone system (RAAS) [66,81].

COVID-19 pathophysiology is largely attributed to dysfunction of innate and adaptive immune responses to SARS-CoV-2 that lead to inflammation, delayed viral clearance, and tissue damage not restricted or localized in the lungs, but systemically affecting other organs with the potential for multiorgan failure [82,83]. Lymphopenia in the blood, a condition of lower-than-normal number of lymphocytes such as T cells, B cells, and innate lymphoid cells is one of the hallmarks of COVID-19 [84,85]. Increased aberrant activation and recruitment of myeloid cells in COVID-19 also may contribute to immune pathology [86,87,88]. Patients with severe COVID-19 have increased circulatory inflammatory cytokines associated with acute lung injury [89,90]. Inflammatory cytokines and chemokines are highly expressed in the bronchoalveolar lavage (BAL) fluid of COVID-19 compared with blood in patients with severe COVID-19, suggesting continuous exposure to viral influence in the lung microenvironment and resulting in heightened local inflammatory status [91].

Host immune response to SARS-CoV-2 plays a critical role in disease pathogenesis and clinical manifestations. SARS-CoV-2 activates antiviral immune responses and causes uncontrolled inflammatory responses with marked pro-inflammatory cytokine release in severe COVID-19 cases, leading to granulocyte and monocyte abnormalities, lymphopenia, and lymphocyte dysfunction. These SARS-CoV-2-induced immune abnormalities may lead to microbial infections, septic shock, and severe multiple-organ dysfunction [83].

Interactions of the SARS-CoV-2 virus with the angiotensin-converting enzyme 2 (ACE2) receptor to gain entry into cells appears to be the predominant mechanism in most organ systems since ACE2 mRNA is present in almost all human organ systems. ACE2 is present in the brain, lung alveolar epithelial cells, arterial smooth muscle cells in the lungs, arterial and venous endothelial cells, kidney parietal epithelial cells, stomach, small intestine, colon, lymph nodes, liver bile ducts, and the skin. However, tissues of the upper respiratory tract tissues (oral, nasal mucosa, and nasopharynx) do not show surface expression of ACE2 on epithelial cells, and thus have less susceptibility to SARS-CoV-2 infection [92]. Multi-organ tissue injuries are linked to altered ACE2 expression and imbalances between expression of the ACE2/angiotensin-(1–7)/mitochondrial Ang system (MAS) and renin–angiotensin system (RAS) in COVID-19 patients [93]. Thus, COVID-19 is characterized as a devastating disease that targets multiple organs because it causes acute complications via organ-specific pathogenesis that ends with the destruction of almost universally present ACE2+ cells. Although the precise pathogenesis of multi-organ damage in COVID-19 is still obscure, the mechanism certainly involves altered ACE2 expression linked with direct/indirect damages due to virus-induced immune responses, such as cytokine storm.

Certain pre-existing health conditions that increase hazard ratios of COVID-19 mortality are considered molecular risk factors possibly due to dysregulated gene expression, in these cases leading to COVID-19-related deaths [94]. Thus, the magnitude of the gene dysregulation is correlated with severity of COVID-19 outcome. These molecular risk factors are potential prognostic biomarkers and targets for therapeutic intervention. Among genes differentially expressed in multiple health conditions, there were 70 upregulated genes and 181 downregulated genes correlated with hazard ratios of COVID-19 mortality, including genes enriched with endoplasmic reticulum, interferon production, mitochondria function, proinflammatory reaction, those involved in programmed cell death, suppressed autophagy that clears virus-infected cells, escalated apoptosis, and necroptosis to eliminate infected cells that participate in viral replication and innate immune responses to viral infections [94,95,96,97].

#### 2.3.3. Long COVID-19 Effects of Post-Acute-COVID-19 Syndrome Sequale

The wide ranging effects of COVID-19, caused by severe acute respiratory syndrome coronavirus 2 (SARS-CoV-2), is manifested in two phases: (1) an acute phase (generally 4 weeks after onset) and (2) a chronic phase (>4 weeks after onset), both of which include a wide variety of physical, neurological, and psychiatric symptoms [98]. The well recognized existence of chronic COVID-19 symptoms, also referred to in various layman and medical vernacular as Long COVID-19, Post Acute COVID-19 Syndrome (PACS), and Post Acute Sequale Coronavirus (PASC), have provided a variety of long-term symptomologies associated with delayed or prolonged physiological effects on host organ systems. Many of these physiological effects are different from those that occur during the acute phase of COVID-19. Long COVID-19 syndrome affects persons of all age groups and is associated with substantial reductions of quality of life [99].

The specific pathophysiological effects and disease mechanims of PASC have not yet been fully clarified, so there are no well-defined criteria or symptomologies specified for the condition. As a consequence, the World Health Organization’s (WHO) definition for PASC is quite broad and it is therefore difficult to confidently diagnose PASC without adequate criteria. The range of PASC symptoms may include general weakness or fatigue, alopecia, amnesia, anxiety, depression, dysosmia, dyspnea, fever, gastrointestinal problems, hypertension, loss of concentration, numbness, pain, palpitation, and sleeping difficulties [98]. Up to 50% of patients may show at least one PASC symptom within one year after acute COVID-19 infection, but the exact occurrence frequencies for individual PASC symptoms has not been determined. The WHO estimates that about 25% of individuals with COVID-19 continue to experience symptoms 4–5 weeks after acute diagnosis and 10% have continuing symptoms after 12 weeks [100].

The difficulty in diagnosing PASC is an ongoing medical challenge that requires more specific information such as PASC-specific VOC biomarkers as well as quantifying SARS-CoV-2 antigens, cytokine levels, and inflammatory markers in plasma samples. Sorting out the complex biochemical processes of PASC and interactions with host metabolic pathways relies on the identification of specific biomarkers that enable classification of patient complex phenotypes (symptomologies) [101]. Patterson et al. [102] analyzed B-cell, T-cell, and monocytic subsets in both severe COVID-19 patients and in PASC patients. They found levels of intermediate (CD14+, CD16+) and non-classical monocytes (CD14Lo, CD16+) were significantly elevated in PASC patients up to 15 months post-acute infection compared with healthy controls (*p* = 0.002 and *p* = 0.01, respectively).

Other definitions of PASC have included persistence of disease >28 days following onset of symptoms, observed in 27–80% of convalescent individuals [103]. In these cases, symptoms range from general fatigue, brain fog, dyspnea, and joint pain to multiorgan impairments [66]. Apart from acute manifestations of COVID-19 illness, increasing evidence points to the development of chronic pulmonary and extrapulmonary effects [104]. Chronic pulmonary effects of PASC often cause diminished lung function and capacity in addition to radiological anomalies, including atelectasis, persistent inflammation, ground-glass opacities, reticulation, and fibrotic-like changes. [71,105]. Extrapulmonary manifestations frequently observed have included myocardial injury, neuropsychiatric symptoms, and thrombotic complications [66,103,105]. Long-term persistence of SARS-CoV-2 viral remnants have been observed in the brain, kidneys, lungs, and gut, possibly by instigating aberrant immune responses [106]. Longitudinal PASC studies have revealed sustained immune response dysregulation of T-cells, highly activated myeloid cells, elevated proinflammatory cytokine levels, and reduction in naive T- and B-cells [107,108,109,110]. In independent PASC cohort studies, a sustained reduction in circulating cortisol immunosuppressive factor has also been reported [111,112].

Possible disease mechanisms involved in Long COVID-19 syndrome may include chronic inflammation, endothelial dysfunction, metabolic perturbations, and gut dysbiosis [99]. Some of these pathogenic mechanisms involving immunologic and systemic effects likely overlap with those of the aging process and may aggravate pre-existing degenerative conditions such as cognitive decline and sarcopenia [113,114,115]. Viral persistence is associated with alterations in immunometabolic pathways, autoimmune processes, chronic inflammation, dysbiosis, endothelial damage, and unresolved organ damage [71,116,117]. Other potential contributors to PASC pathogenesis and symptom longevity may include consequences of disease-related injuries to multiple organs, persistent SARS-CoV-2 reservoirs in certain tissues, reactivation of neurotrophic pathogens (herpesviruses etc.) during COVID-19 immune dysregulation, SARS-CoV-2 effects on host microbiome/virome communities, clotting and coagulation issues, dysfunctional brainstem and vagus nerve signaling, activities of primed immune cells, and autoimmunity [118]. The multifactorial pathophysiology of PASC, causing a multiorgan disease with broad spectrum of manifestations, also has been proposed to involve immobility and metabolic alterations during critical illness and microvascular ischemia and injury [71,119].

## 3. Identification of COVID-19-Specific Chemical Biomarkers

Metabolic processes in healthy individuals maintain a balanced system of homeostasis that give rise to normal volatile metabolites which become dissolved in the blood, and if sufficiently volatile, are released from the body via the lungs through the human breath. The normal or healthy state metabolome consists of VOCs components that are released in the human breath at concentration (levels) indicative of a healthy state within certain ranges and molar ratios. When the levels of breath VOCs change from normal health ranges, which vary with age, race classes, and other factors, this is an indication of possible disease developing at early stages of pathogenesis. By virtue of the circulatory system that links and transports volatile metabolites from all organs throughout the body to the lungs, one cannot assume that VOC metabolites, expelled in breath from lung tissue, originated from metabolic pathways occurring in the lung itself or from other organs of the body. This problem precludes easy identification of the specific metabolic pathways giving rise to volatile metabolites released in exhaled breath without the use of additional techniques such as radioactively C-labeled metabolites.

Altered changes in metabolic processes not only arise from the effects of infectious diseases on human tissues, but also development because of other major health problems such as metabolic diseases, including diabetes mellitus, dyslipidemia, osteoporosis, and obesity which are often due to an unhealthful lifestyle or poor diet [120]. Recently, analyses of breath VOCs, particularly short-chain fatty acids (SCFAs), have been considered useful tools for mechanistic understanding and diagnosis of metabolic diseases, especially those associated with poor diet or microbial dysbiosis of the gut microbiome [121].

Metabolomics is now considered a powerful approach for disease biomarker discovery associated with metabolic dysregulations. However, the diagnosis of diseases based on metabolomics is difficult and cumbersome when based on breath analysis alone because this diagnostic approach involves measuring altered levels of key physiological metabolite biomarkers occurring in metabolic pathways in various organs and tissues distally separated from the lungs. The biggest problem is the difficulty in determining the originating distal sources (organs, tissues, and associated metabolic pathways) involved in generating VOC disease biomarkers identified in exhaled breath. This work typically requires the use of radioactively labeled metabolites or metabolite precursors introduced into diseased patients, through ingestion or injection, to detect and identify the physical sources and metabolic pathways from which these labeled metabolites originated before passing through the circulatory system to the lungs. One such example is the use of radioactively labeled carbon, ^13^C-labeled prebiotic inulin, fed to subjects and microbially fermented in the human gut to produce ^13^C-labeled short-chain fatty acids which were detected as having higher plasma acetate concentrations compared with controls, and increased ^13^CO_2_ enrichment in the human breath following translocation of metabolite intermediates through the circulatory system [122].

Research into the metabolomics of COVID-19 pathogenesis has revealed numerous metabolic pathways affected by the disease, which may indirectly help explain many quantitative changes in certain VOCs levels present in the breath of COVID-19 patients. Most of the COVID-19 biomarker metabolites identified hitherto from metabolomic studies of various organ systems are nonvolatile and thus of little use in COVID-19 disease detection via breath analysis. The quantitative nonvolatile metabolite biomarkers identified from various organ systems are metabolites that have already been well associated with metabolic pathways occurring in specific organ systems. However, determining differences in VOCs present in exhaled breath of COVID-19 patients vs. non-COVID-19 individuals using metabolomic chemical-analysis techniques, such as GC-MS and NMR, does not strictly qualify as metabolomic studies unless this breath VOC information can be directly linked to specific metabolic pathways (distal to lung tissue) affected by COVID-19 pathogenesis.

Determining the specific metabolic pathways in the body that are being affected by pathogenesis gives strong clues as to the pathogen involved and the likelihood of which VOCs will change due to disruptions of specific metabolic pathways [18]. Investigations into detailed information concerning pathogenesis and the pathophysiological mechanisms of disease, especially in specialized area of cellular metabolomics, provide more details about how a particular disease affects and diverts normal metabolic processes in the body to generate an abnormal disease state.

### 3.1. COVID-19 Induced Quantitative Changes in Volatile Metabolites Released in Breath

A firm understanding of the pathophysiological mechanisms by which the SARS-CoV-2 virus affects the body at the chemical level is essential for identifying candidate COVID-19-specific volatile disease biomarkers as potential VOC metabolic targets for disease detection. As with all infectious diseases, the mechanisms by which pathogenic agents cause disruptions of normal cellular metabolic pathways to induce host cellular responses, and ultimately changes in cellular metabolites, are unique to individual microbial groups [18]. Pathogenesis-induced changes in metabolic pathways produce volatile metabolites which are carried through the circulatory system and are ultimately released from the lungs via exhaled breath [16,123]. The chemical effects of COVID-19 on cellular metabolism are first initiated by a multistep process at the cellular level, beginning with viral attack at the cell surface, leading to virus entrance into cells to initiate disruptive controls and redirection of normal cellular metabolic processes to new processes involved in virus replication. The resulting wide variety of possible metabolic effects of COVID-19 are determined by the specific types of tissues being infected and the unique chemistry of metabolic processes that occur in the specialized tissues of different organs involved.

At least five major pathophysiological mechanisms have been identified to explain the key pathogenesis processes involved in COVID-19 systemic effects and SARS-CoV-2 virus–host interactions, including (1) SARS-CoV-2 spike protein interacting with angiotensin-converting enzyme 2 (ACE2) on the cell surface of epithelial cells to induce cytotoxicity, (2) dysregulation of the renin–angiotensin–aldosterone system (RAAS) as a result of virus-mediated ACE2 downregulation, (3) dysregulation of immune responses causing acute inflammatory responses elicited with overproduction of pro-inflammatory cytokines and chemokines, (4) endothelial cell injury leading to activation of the coagulation cascade with intravascular thrombo-inflammation, and (5) extensive tissue destruction, interstitial thickening, fibroblast proliferation, and tissue fibrosis [66]. This list does not include the many additional detailed mechanisms by which COVID-19 disrupts the normal processes of many other metabolic pathways that occur in different organ systems throughout the body. Viral disruptions of these many metabolic pathways generate both volatile and nonvolatile metabolites, depending on the molecular weight of metabolite intermediates that occur within any given metabolic pathway affected. The following sections summarize some of the effects of COVID-19 on the metabolic pathways of specific organs, but focus mostly on SARS-CoV-2’s viral effects causing dysregulation of sometimes currently unknown metabolic pathways that produce quantitative changes in volatile metabolites (i.e., VOCs), which are the only ones of interest as potential biomarker targets for COVID-19 detection in human-exhaled breath. The effects of COVID-19 on metabolic pathways giving rise to nonvolatile metabolites, detected in blood serum and plasma and other biological fluids, are discussed in more detail under Section 6, titled Metabolomic research of nonvolatile biomarkers.

Most breath-analyses studies to identify COVID-19-specific volatile metabolites to date have not thoroughly investigated the actual sources of these volatile biomarkers and have only documented quantitative differences between sample types (from healthy and diseased individuals) and correlated this data for the purposes of COVID-19 diagnostic determinations. Despite the existence of only limited studies and information to help identify the specific mechanisms by which volatile metabolites found in the exhaled breath of COVID-19 patients are generated, some basic information has provided clues to possibly explain at least some of the metabolic pathway dysregulation that leads to quantitative changes in breath VOC concentrations. Individuals with COVID-19 often have exhibited higher levels of aldehydes and ketones in their exhaled breath. Greater production of aldehydes often may occur when tissues are damaged by inflammation [124]. COVID-19 induction of systemic cytokine storms, with associated metabolic cascades, affects many organs and cause widespread cellular damage, acute inflammation of tissues, and immunosuppression. Increases in breath ketones indicate that the SARS-CoV-2 virus damages the pancreas and liver, worsening complications of diabetes, disrupting key metabolic signals causing symptoms of ketosis, hyperglycemia, or hypoglycemia due to effects on insulin and glucose metabolism [125,126]. COVID-19 often causes comorbidity effects that result in worsening preexisting diseases that exist in the body prior to SARS-CoV-2 infections. Preexisting hyperglycemia can contribute to SARS-CoV-2 increased entry and replication [127]. Individuals with obesity and related metabolic diseases, such as type 2 diabetes (T2D), typically have more devastating COVID-19 disease courses. COVID-19 pathogenesis mechanisms and the dysregulation of host metabolism are intimately connected, although the details of the molecular mechanisms of deregulated glucose metabolisms that determine vulnerability of cells and organs in a diabetic environment are not well understood.

Other known COVID-19 VOC metabolic sources, such as certain aldehydes and alkanes derived from lipid oxidation and ketones from carbohydrate and fatty acid metabolism, may help explain some of the indirect up-regulation due to COVID-19 pathogenesis [128,129]. The causes of up-regulation in certain alcohols (ethanol, propanol, and butanol), but down-regulation of methanol are not yet fully explained. The up-regulation of ethanol is likely associated with COVID-19 effects on acetaldehyde metabolism that occurs primarily in the liver, resulting in increases in blood concentrations of this alcohol, possibly causing alcohol intoxication-like symptoms in severe cases. The breakdown products and derivatives of these COVID-19 induced dysregulated low molecular weight VOC metabolites, originating from many endogenous metabolic processes, also are carried through the circulatory system and rapidly excreted from the lungs.

Human exhaled breath typically contains thousands of VOCs that are a culmination of intermediate and end products of many metabolic pathways occurring throughout the body. The sensor array of e-nose devices collectively responds proportionally to all VOCs present in a breath sample (for which individual sensors are sensitive), with individual sensors responding variably as indicated by a net sensor response intensity. Thus, e-nose devices do not identify individual VOCs in the sample. Not all, but only a smaller subset of VOCs present in breath samples are identified as COVID-19 disease biomarkers. A breath VOC is not recognized as a COVID-19-specific biomarkers unless it is consistently established and highly correlated with the presence of the disease only in SARS-CoV-2 infected individuals as confirmed by many independent studies. The associated chemical classes of individual VOC biomarkers are usually determined by metabolomic studies first, providing clues as to the likely types of metabolic pathways being affected by disease processes.

Some researchers have used and defined the term ‘biomarker’ incorrectly by extending the definition to include groups or clusters of VOCs that collectively are referred to loosely as a composite biomarker, usually based on complex statistical models developed to discriminate between clinical sample types. Chemical biomarkers largely lose their meaning and effectiveness as diagnostic tools, in many cases, when they are aggregated into cluster groupings. By contrast, the diagnostic value of well-established individual chemical biomarkers, identified by metabolomics, can strongly support diagnoses based on breath e-nose analyses. The more individual disease VOC biomarkers detected in co-analyzed (e-nose + metabolomic method) breath samples as being dysregulated (quantitatively changed in concentration relative to controls) or uniquely present (qualitatively unique to SARS-CoV-2 infections), the stronger the confirmation and diagnostic value of biomarkers detected. Thus, the statistical certainty and confidence of e-nose based diagnoses generally increases exponentially as more disease biomarkers are detected in the same samples by metabolomic methods [16,18,123]. This principle supports the combined use of both e-nose and metabolomic methods, or dual-technology instruments containing e-nose and chemical-analysis capabilities, when necessary for diagnostic confirmations.

An early study by Ruszkiewicz et al. [48], who analyze the breath VOC profile signature of inflammatory and oxidative stress metabolites detected from COVID-19 positive patients (compared to COVID-19 negative patients) using gas chromatography and ion mobility spectrometry (GC-IMS), identified the following ten VOCs (in alphabetical order) as possible quantitative disease biomarkers: acetone, alcohol, butanone, heptanal, isoprene, methanol, octanal, propanal, and propanol along with one unidentified metabolite (Table 2). The SARS-CoV-2 virus caused an upregulation in the production of nine volatile metabolites, resulting in increases in VOC concentrations of these components in the human breath relative to healthy controls, and a downregulation of only methanol that decreased in concentration relative to controls. The chemical classes of these possible COVID-19 quantitative biomarkers included four aldehydes, two ketones, two alcohols, and one alkadiene or hemiterpene.

Most other metabolomic studies investigating COVID-19 induced breath volatiles also found various aldehydes as the predominant chemical class representing VOC components that increased in concentration relative to controls [23,131,132]. All references detected at least one aldehyde except for Chen et al. [130] who detected a ketone (acetone) that decreased in concentration, but increases occurred for alcohol and ten other unidentified metabolites. Alkanes and alcohols also were significant VOC components found to increase in the breath of COVID-19 individuals [48,131,133]. Berna et al. [132] indicated that heptanal was identified as the most consistent and key VOC biomarkers elevated in SARS-CoV-2 infected patients.

Some unusual minor volatile metabolites were found in some studies to be present and increasing in the breath of COVID-19 positive individuals including isoprene and 2,4-octadiene (both alkadienes), propionic acid (a carboxylic acid), 2-pentyl furan (a furan derivative), benzaldehyde (an aromatic aldehyde), camphene (a bicylic monoterpene), and β-cubebene three terpenes (a tricyclic sesquiterpene) [131,132,133]. Some of these minor metabolites may actually turn out to also be qualitative biomarkers of COVID-19, but occur at sufficiently low concentrations due low levels of upregulation that they are nondetectable in many COVID-19 individuals. A few VOCs present in COVID-19 such as 1-chloroheptane (a chloronated alkane) and iodobenzene (an organoiodine benzene derivative) were halogen-containing hydrocarbons that were probably artifacts in the analysis because they are not normally produced endogenously [23,133].

Laird et al. [134] used the AtmosFTIR platform to determine that higher concentrations of methanol, ethanol, and acetaldehyde were found in the exhaled breath of the COVID-19 group with symptomatic patients being higher than in SARS-CoV-2 infected asymptomatic patients. In addition, they found that carbon dioxide levels decreased in SARS-CoV-2 infected symptomatic patients compared to infected asymptomatic and control patients. These results are consistent with increases in alcohols and aldehydes found in COVID-19 patients. The occurrence of the lowest CO_2_ concentration in symptomatic patients indicated that this may be linked to the severity of the disease and ability to breathe.

Liangou et al. [131] detected an increase in the inorganic gas nitric oxide (NO), also known as nitrogen monoxide, in the breath of COVID-19 individuals. Gould et al. [135] found that NO production was associated with oxidative stress caused by many types of viral infections. However, Nikolaidis et al. [136] concluded that the comorbidities associated with negative clinical patient outcomes of COVID-19 sickness are linked to depletion of NO in the body.

The presence of NO in the breath is normally considered a strong diagnostic indicator of asthma and NO levels in the breath are commonly used to detect the severity of asthma effects on the lungs [137]. These observations suggest that the mechanism by which the SARS-CoV-2 virus attacks the lungs could be similar to the effects of asthma. NO is generated during conversion of the amino acid L-arginine to L-citrulline by nitric oxide synthase (NOS) using NG-hydroxyl-L-arginine as an intermediate [138]. L-arginine is a common substrate of both nitric oxide synthase and the arginase pathway. NO nitric oxide is produced by constitutive and inducible nitric oxide synthases which have pathophysiological important roles via direct or indirect effects on oxidative stress production.

The importance of NO in the pathogenesis of COVID-19 was elucidated in a review by Ranjbar et al. [139]. NO is a vasodilator produced by endothelial cells which could potentially be used to treat COVID-19. NO synthesis and release are promoted by Ang-(1–7), a metabolite produced by ACE. Since SARS-CoV-2′s spike protein binds to ACE2, it affects the production of NO by decreasing production of Ang-(1–7) [140]. NO is able to inhibit the COVID-induced cytokine storm, improve oxygenation of the arteries, and inhibit pulmonary hypertension by its vasodilatory actions within the lungs [141]. These findings suggest that patients could benefit from exogenous NO because of its anti-inflammatory effects. Because NO now has been associated with protective measures, especially for ARDS patients resulting from COVID-19 virus infections, the prescription of NO treatments for COVID-19 patients is justly warranted. Supplemental NO administered through inhaled nitric oxide or donor compounds could help combat the detrimental effects of this virus [142]. The NO donor, S-nitroso-N-acetylpenicillamine (SNAP) is able to interfere with the fusion of the SARS-CoV spike protein with its receptor and diminish the virus’ RNA synthesis [143]. The increased severity of COVID-19 symptoms associated with old age and the age-related decline of NO levels have tied low NO levels to all major high-risk groups of COVID-19 infection. This correlation of low NO levels to increased COVID-19 infection severity and COVID-exclusive symptomatology, along with successful utilization of NO gas as a therapeutic option, should stimulate further research on this basis to further improve COVID-19 treatment options [136].

Respiratory viral infections induce the release of inflammatory cytokines in the body which result in the production and release of nitric oxide (NO), ammonia (NH_4_), and VOCs. Besides GC-MS gas detection, certain nanosensor systems also are capable of detect these exhalents and have the potential for early detection and disease monitoring of patients with viral respiratory infections. The specific targeting of volatile inorganic, nonVOC-type chemical biomarkers, known to be associated with SARS-CoV-2 infections, provides and alternative approach using of a single selective, resistive chemosensor. Exline et al. [144] developed a novel breathalyzer electronic technology that utilizes a single sensor made of a catalytically active, semiconducting sensing film, produced by means of sol-gel processing using tungsten alkoxide precursors, that targets NO and ammonia detection in breath within 15 s. The single sensor allows for rapid analysis and diagnostic results. The sensitivity of the γ-phase tungsten trioxide (WO_3_) sensor to NO, selectivity and response in the presence of various interfering compounds have been demonstrated previously [145]. The specificity of the instrument for detecting COVID-19 is based on the production of a distinctive pattern or breathprint for patients with active COVID-19 pneumonia. The breathprint identified COVID-19 pneumonia patients with 88% accuracy upon admission to the ICU. The sensitivity index of the breathprint, scaled with the key biomarker ammonia concentration, correlated with duration of the SARS-CoV-2 infection.

### 3.2. Disease-Specific Qualitative COVID-19 Biomarkers

The previous section demonstrates that the vast majority of COVID-19-specific breath VOC biomarkers identifed so far are of the quantitative or metabolomic type in which the levels of normal breath VOCs produced and released in healthy individuals are either increased or decreased due to COVID-19 pathogenesis. Investigations into the possible existence of qualitative COVID-19 disease-specific VOC biomarkers produced only in diseased individuals with SARS-CoV-2 infections, but not produced in healthy or non-COVID-19 individuals, have only just begun and much more research is still required. One possible example of a qualitative COVID-19 biomarker was detected by Ruszkiewicz et al. [48] who found a large, unidentifed VOC peak in a 3-D GC-IMS plot of data acquired by breath analysis and comparisons from 98 COVID-19-positive and -negative patients located at two hospital sites in Edinburgh, UK and Dortmund, Germany. They identified specific biomarkers distinguishing between the two groups used in the final PCA Modeling. They found one of the VOC peaks (M7 or Feature 144) was not yet identified from the GC-IMS reference database. Other diagnoses among the cohort included asthma, COPD, bacterial pneumonia, and cardiac conditions. The use of multivariate analysis identified two aldehydes (ethanal, octanal), two ketones (acetone, 2-butanone), and methanol that discriminated COVID-19 from the other conditions. Other VOC biomarkers among the best discriminants between COVID-19-positive and COVID-19-negative patients included isoprene, propanal, and propanol, and heptanal. The unidentified M7-VOC isolated in Edinburgh had very significant predictive power for severity/death, whereas heptanal was identified as most significant in Dortmund. The study concluded that the identity of the nine VOC biomarker compounds were consistent with COVID-19 derangement of breath-biochemistry by ketosis, gastrointestinal effects, and inflammatory processes. More GC-IMS research at multiple other locations will be required to determine if the M-7 VOC is consistent enough to be considered a qualitative biomarker, since it may vary in detecteability depending on different sources and methods of patient air sample acquisition. Other possible qualitative biomarkers may be identified with additional research.

### 3.3. Human Cell-Line COVID-19 Culture Viral Biomarkers

Viral infected human cell cultures have been used extensively in the past to study the release of VOCs associated with previous flu and coronavirus pandemics, which are biosafety level-2 viral agents. However, relatively few studies of this type have been carried out for COVID-19 research into disease biomarkers because of the higher biosafety level of this viral agent. The SARS-CoV-2 virus is classified as a biosafety level-3 (BSL-3) agent, impeding basic research into its biology and development of effective antivirals. Consequently, Ju et al. [146] had to develop a biosafety level-2 (BSL-2) cell culture system for production of transcription and replication-competent SARS-CoV-2 virus-like-particles (trVLP) to study this virus. This trVLP expresses only a reporter gene (GFP), replacing the viral nucleocapsid gene (N) required for viral genome packaging and virion assembly (SARS-CoV-2 GFP/ΔN trVLP). The complete viral life cycle was exclusively confined in the cells ectopically expressing SARS-CoV or SARS-CoV-2 N proteins, but not MERS-CoV N. Genetic recombination of N supplied in trans into the viral genome was not detected from sequence analysis one-month after serial passages in the N-expressing cells.

Several cellular and animal 2-D cell models have been used for studying SARS-CoV-2 infection. In vitro models are useful for studying virus biology under highly controlled conditions, but these models are limited in utility because they often fail to duplicate the complex biochemical interactions occurring in whole human body systems [147,148]. However, in vivo models for SARS-CoV-2 infection have the potential to elucidate some details of COVID-19 pathophysiology, contributing to the discovery of COVID-19 antivirals and vaccines [149], but are costly, requiring BSL-3 animal facilities, and raise valid ethical concerns. Cellular models do not resemble the complexity of a whole organism and thus in vitro data are not translatable to in vivo models [150]. The use of 3-D cell models in SARS-CoV-2 research has gained attention due to closer similarity with the host organism. Organ-derived explants, produced by induced stem cells, are now widely applied to the study of respiratory viral infections [151]. Results obtained from specific tissues are important for determining possible responses to viral infection and finding new therapeutic targets. Patient-derived explants have the advantage of preserving overall tissue architecture and complexity compared with organoids, but organoids may be more easily standardized.

### 3.4. Application and Uses of COVID-19-Specific Chemical Biomarkers

Samprathi et al. [49] recently listed nine ways in which identifying COVID-19-volatile and -nonvolatile biomarkers could be useful in more effectively dealing with decisions relating to treatment of infected patients, including early detection of the disease (confirming infection status), confirmation and classification of disease severity, defining and implimenting hospital and ICU admission criteria, identification of high-risk cohorts, rationalizing appropriate therapies, assessing response to therapies, developing prognoses, and establishing criteria for discharge from the ICU and/or hospital.

## 4. Experimental and Clinical COVID-19 Detection by E-Noses

Research involving the development of e-nose technologies for COVID-19 detection was initiated soon after the pandemic began, and the SARS-CoV-2 virus started spreading globally in the early months of 2020. The efficacy testing of new experimental and commercially available electronic-nose devices for potential detection of SARS-CoV-2 infections, based on different applications and approaches for disease detection at various stages of COVID-19 development, have utilized a variety of e-nose sensor arrays with variable sensor numbers and sensor types (Table 3). Most of the seven e-nose devices tested so far are of the MOS sensor type and contain 10 or less MOS sensors. The few uniquely different sensor array type instruments include the portable Cyranose 320 e-nose device with 32 carbon black polymer composite (CBPC) sensors and the NaNose with 8 gold nanoparticle (GNP) sensors. All except one of these devices had preexisted with earlier developmental histories prior to the occurrence of the COVID-19 pandemic and were not originally developed specifically for COVID-19 detection but had been tested for use in detecting other respiratory diseases and cancers. The one exception is the newer experimental e-nose device developed by Kwiatkowski et al. [152] that consists of a sensor array composed of three micro-electro–mechanical system (MEMS) sensors and a single MOS sensor. MEMS is a chip-based technology in which the sensors are composed of a suspended mass between a pair of capacitive plates that creates a difference in electrical potential when the sensors are tilted, which is measured as a change in capacitance. By contrast, MOS sensors detect various types of gases by measuring the electrical resistance change that is induced when the gases adsorb to the sensor surface coating. As with MOS sensors, different GNP sensors have a diverse sensing layer on the sensing surface that swells or shrinks upon exposure to different types of VOCs, causing changes in electric resistance. GNP sensors are multilayered and are composed of gold nanoparticles linked to different kinds of organic ligands with an organic film element that provides sites for the adsorption of VOCs and an inorganic nanomaterial layer responsible for changes in electrical conductivity. [153,154]. When the sensors are exposed to VOCs, the gases adsorb onto or diffuse into the sensing layer and react with the organic segment, having exposed the functional groups that cap the inorganic nanomaterials.

The MOS sensor types used in most e-nose tests for COVID-19 detection applications generally operate at higher temperatures and their application is limited to situations where there is a readily available power source at the site of breath air sample analysis. Consequently, MOS-sensor-based e-noses often are not as useful in outdoor field situations for real-time diagnostic applications where mass sampling is carried out, such as in drive-by testing centers. In these situations, e-nose devices designed for operating a lower or room temperatures may be more effective without the availability of electrical power sources. Alternatively, breath air samples may be acquired from individuals using special VOC-retentive air bags for quick analysis later at indoor sample-processing facilities.

The handheld portable electronic nose Aeonose, developed by Aeonose Company in Zutphen, the Netherlands, was tested as a pre-operative screening tool for the detection of SARS-CoV-2 infections to distinguish COVID-19-positive from -negative participants based on VOC patterns in exhaled breath with a high negative predictive value (NPV) of 0.92 [155]. This e-nose consists of eight micro hotplate MOS sensors and carbon monoxide (AS-MLC), nitrogen dioxide (AS-MLN), and VOC (AS-MLX) sensors. Breath air sample types were obtained directly into the e-nose without a special mouthpiece device for filtering particulates or other contaminants. The use of eight MOS sensors for VOC discrimination in addition to the single AS-MLX VOC sensor provides adequate power for analyzing differences in volatile organics, but this is also augmented by two non-VOC sensors that measure the dysregulation of other gases frequently associated with COVID-19 patients. The reduction in output of both CO and nitrogen dioxide gases in exhaled breath associated with generally reduced lung capacity indicate additional, measured lung gas flux parameters to help confirm sensor response pattern changes in VOC emissions. The advantages of adding additional sensors to measure inorganic gas exchange emissions are confirmed by known effects of COVID-19 on these emissions as co-factors in diagnoses.

The study included 219 participants, 57 of which were COVID-19-positive and 162 were COVID-19-negative. The Aeononse breath test failed due to dyspnea or technical difficulties in only 3% of the samples. The Aeonose may be a useful noninvasive and promising low-cost triage tool for excluding SARS-CoV-2 infection in patients elected for surgery. Important reasons for e-nose screening are that COVID-19 patients have increased risk for adverse postoperative outcomes from aerosol exposures of other hospitalized infected patients and hospital workers. COVID-19-positive pre-operative patients might be rescheduled to avoid these risks or necessary precautions could be taken to limit chances of transmission [161].

An exploratory clinical study, initiated in Wuhan China in March 2020 during the early months of the COVID-19 pandemic near its origination, was conducted using a cohort that included 49 confirmed COVID-19 patients, 58 healthy controls, and 33 non-COVID-19-infected lung infection controls [44]. The NaNose e-nose, consisting of eight NGP sensors in the array, was integrated with electronic circuitry to an advanced apparatus that collected exhaled breath samples from subjects blowing air into the device for 2–3 s from a maximum distance of 1–2 cm. A built-in sensor system composed of a nanomaterial-based hybrid sensor array with multiplexed detection capabilities provided a means to detect COVID-19 disease-specific biomarkers from exhaled breath, enabling rapid and accurate diagnosis. The device contained sensors with surface-sensing layers within the organic segment with specific organic functionalities (exposed functional groups) including dodecanethiol, 2-ethylhexanethiol, 4-tertmethylbenzenethiol, decanethiol, 4-chloro-benzenemethanethiol, 3-ethoxythiophenol, tert-dodecanethiol, and hexanethiol capable of the detection of possible VOC breath biomarkers. Study results showed an AUC of 0.81 (95% CI, 0.70 to 0.89) in patients with COVID-19 vs. with controls. A higher AUC of 0.97 (95% CI, 0.92 to 0.99) was observed in COVID-19 vs. other lung infection/conditions, and an AUC of 0.87 (95% CI, 0.67 to 1.00) for COVID-19 first samples vs. COVID-19 sample tests. Significance of the results (*p* < 0.001) was found for the comparisons of the training set for each of binary classification. The high-functioning of this e-nose attests to the proper selection of specific sensors in the sensor array that were appropriately determined to be effective in the design phase for applications in detecting viral respiratory diseases. The extended application to COVID-19 detection was fortunate and efficacious based on current performance data.

Kwiatkowski et al. [152] developed an experimental e-nose composed of three MEMS sensors and 1 MOS sensor, all commercially available VOC gas sensors, to fill the need for a relatively cheap POCT device for mass daily screening of large crowds in airports, schools, and stadiums where rapid noninvasive methods are required which also deliver fast results. The exploratory study utilized patients from a local hospital to test the efficacy of the methods and device to help limit the spread of COVID-19 and similar viral infections. The breath samples were collected using a BioVOC sampler from COVID-19-infected patients during the third wave of the pandemic in Poland between March and July 2021 when SARS-CoV-2 Alpha and Delta variants dominated within this period in Poland. The test included hospitalized COVID-19 patients and a control group from which 56 breath samples were taken (33 patients with severe course of COVID-19 disease, 17 breath samples collected within the healthy control group, and 6 samples of ambient air). Discriminating models presented divergent results with AUC above 0.83 for these algorithms for both cohorts. Discrimination between COVID-19-infected and healthy patients only resulted in 84% classification accuracy for the Neural Network and Random Forest algorithms. Worse results of 70% classification accuracy were observed for the KNN algorithm. Exclusion of humidity sensor data resulted in a drop in classification accuracy to 66% for the KNN algorithm only. The other algorithms secured better classification accuracy between 70 and 74%. COVID-19 infection was more severe for elderly patients who had statistically greater changes in exhaled breath than for younger patients over time with disease progress. The cohort was divided into two subsets using as a threshold the median age of COVID-19-infected (55 years) and healthy volunteers (45 years) analyzed separately. The evaluated ROC curves confirmed better e-nose detection results for the older group with a classification accuracy over 91% for all algorithms. However, the detection accuracy dropped to 80% for the younger group of patients in the worst-case neural network algorithm. These results endorse the proposed methodology for use with an e-nose that is statistically more effective in exposing the most vulnerable elderly patients. The lower performance level of this e-nose is largely due to the minimal number of sensors selected in the sensor array that do not effectively discriminate between complex VOC mixtures, associated with different sample types, because of reduced permutations possible in smellprint signature combinations. Many variables associated with changes in VOC compositions in breath samples can reduce the effectiveness of sample discrimination power if the sensor arrays lack sufficient sensor numbers to cover the wide variation of VOC types encountered in air sample analytes.

The commercially available compact PEN3 eNose (AIRSENSE Analytics GmbH, Schwerin, Germany), containing a 10 MOS sensor array, was used by Snitz et al. [156] in combination with a one-way disposable intranasal sampling valve to acquire subject air and a canister of pressurized medical grade air to continuously inflate a large breathing bag that served as clean reference air for the eNose. The apparatus was tested at a national testing station ran by Magen David Adom in Tel Aviv, Israel where people were sent via a national referring system that assigned tests to individuals who had a lengthy exposure to a verified COVID-19 patient and/or were experiencing persistent COVID-19 symptoms. They obtained a mean AUC of 0.63, with a true positive rate ranging from 47.4% to 94.4% and mean of 75.8% with this apparatus. The researchers concluded that additional e-nose improvements and results were possible by optimizing the sensor coating specificity, based on analysis of COVID-19 volatile biomarkers, to provide effective real-time diagnoses in locations such as airports, the workplace, and cultural events. The authors acknowledge the need to modify the composition of sensor coatings to improve targeting of COVID-19-specific volatiles to increase sensitivity and improve diagnostic performance.

Bax et al. [157] developed breath aroma fingerprints from exhaled breath samples, assessed using a commercial EOS-AROMA e-nose with a custom 4-MOS sensor array in combination with a T-piece valve and 5L NalophanTM bags as a system to help the management of respiratory failure patients. They carried out a feasibility study on 33 SARS-CoV-2 patients, consisting of 25 with respiratory failure, 8 asymptomatic, and 22 controls to gather data on tolerability to the breath analysis apparatus and for a preliminary assessment of sensitivity and specificity. The most significant features for discrimination of breath aroma fingerprints from respiratory failure patients vs. controls were identified using the Boruta algorithm and support vector machine (SVM) classification model. The SVM differentiated between respiratory failure patients and controls with an AUC of 0.81, sensitivity of 0.920, and specificity of 0.682, respectively. The selected analyzed features were significantly different in SARS-CoV-2 patients with respiratory failure vs. controls and asymptomatic SARS-CoV-2 patients (*p* < 0.001). The developed analysis system was determined to be suitable for exhaled breath collection from respiratory failure patients to obtain diagnostic breath fingerprints useful as sensitive markers of lung disease severity and etiology. This e-nose design is also somewhat compromised by limited numbers of sensors in the sensor array that reduced specificity, accuracy, sensitivity, and performance. An increase in the number of sensors in the sensor array with diverse capabilities of discriminating a wider range of COVID-19-specific VOCs would improve the effectiveness of this e-nose.

The portable GeNose C19 e-nose with 10 MOS sensors was assessed for COVID-19 detection by two independent research groups. Nurputra et al. [158] originally developed and tested the potential of the instrument as a low-cost, noninvasive screening tool to distinguish patients with COVID-19 from other respiratory diseases at the bedside clinical setting. They employed a mask-integrated sampling bag for collecting and profiling 615 breath samples composed of 333 COVID-19-positive and 282 -negative samples obtained from 43 positive and 40 negative COVID-19 patients. They utilized four different machine learning algorithms, including linear discriminant analysis (LDA), support vector machine (SVM), stacked multilayer perceptron (SMP), and deep neural network (DNN) to determine the best-performing pattern recognition methods and obtain a high system detection accuracy of 88–95%, sensitivity of 86–94%, and specificity of 88–95%. Their results suggested that the GeNose C19 device had high potential as a breathalyzer for fast COVID-19 screening. Hidayat et al. [160] tested an instrument with similar accuracy results. They developed a hybrid machine learning-based algorithm that combined hierarchical agglomerative clustering analysis and a permutation feature importance method to improve the data analysis of the instrument. With this this learning approach, they obtained an effective and optimum feature combination which allowed reduction of the number of sensors employed by half without downgrading the classification performance. Based on cross-validation test results, the hybrid algorithm resulted in values for accuracy of 86 ± 3%, sensitivity of 88 ± 6%, and specificity of 84 ± 6%. The effectiveness of using this hybrid filter wrapper and feature-selection method helped to optimize the GeNose C19 instrument’s performance. The use of several statistical sample discrimination models to facilitate distinguishing between clinical sample types improved data analysis and performance of this e-nose. The authors effectively developed machine learning-based algorithms to combine hierarchical agglomerative clustering analysis with permutation features to improve test results.

Nidheesh et al. [159] conducted a pilot study using the Cyranose C-320 e-nose device with a 32-sensor carbon black polymer composite (CBPC) sensor array for point-of-care diagnostic testing of post-coronavirus disease syndrome (PCS). Breath samples of cohorts consisting of PCS, asthma, and normal (control) subjects were tested. Match/no-match and k-nearest neighbors (KNN) algorithm was used to assess the sensor’s performance to discriminate between sample types and to confirm the diagnosis of PCS. Their prediction model yielded 100% sensitivity and specificity with an accuracy for the prediction model AUC of 1.0. The analysis of sensor intensity responses of all sensors in the sensor array indicated that sensor numbers 5, 23, and 31 had significantly higher sensor responses to breath samples from post-COVID-19 PCS individuals than with asthma and normal control subjects. They further determined that the higher sensor responses to breath samples of PCS subjects suggested that the main VOC differences in these samples (compared with asthma and control samples) were most probably due to higher concentrations of aldehydes, alcohols, and ketones in post-COVID-19-positive individuals, based on 32-sensor response data obtained from the C-320 e-nose by running pure analytical grade samples from specific chemical classes according to Doty et al. [162]. These data are consistent with the same VOC biomarker chemical classes identified in metabolomic studies presented previously in Section 3.1. In a similar previous report by Zamora-Mendoza et al. [45] involving breath analysis of COVID, PCS, and control samples analyzed with the C-320 e-nose, a sensitivity of 97.4% was obtained in the classification, confirming the successfully use of this instrument for point-of-care applications in diagnosing PCS. The additional benefits and advantages mentioned for this technique over other usual clinical procedures included non-invasive testing, cost-effective application, need of less expertise to operate, and fast diagnosis (results in <10 min). The C-320 e-nose has several advantages over other portable types including its light weight, high sensor number (n = 32) for effective discriminations, and its tested performance for many human disease-detection applications. The CBPC sensor array can detect VOCs in air samples with high levels of moisture content because the sensor array is not affected as significantly by high relative humidity. Thus, the C-320 e-nose is ideally suitable for breath air samples, although it is best to precondition the sensor array for the moisture content of the samples being analyzed to improve data quality for discriminations. This instrument has a limited number of sample types that may be stored in its memory simultaneously, but this problem could be solved with improvements in software and instrument redesigns to accommodate larger numbers of sample types encountered for diagnostic applications.

## 5. Electronic-Nose Sensor Array and Smellprint Signatures

Electronic-nose devices generally consist of a multisensor array, composed of variable numbers of sensors with differential sensitivities to different classes of VOCs, which produces a sensor response (smellprint) pattern when collectively assembled into a graphic digital output format such as a radar plot or bar graph. The combined sensor–response pattern is a visual representation of the collective sensor array responses to the entire complex of VOC components present in the sample analyte that the sensors are capable of detecting. Some VOC components may not be detected by any of the sensors in the sensor array and therefore these VOCs are not represented by the smellprint signature. This is the reason why e-nose sensor arrays should contain sensors that have specific sensitivities to the full range of VOC chemical classes represented by target compounds expected to be present in the specific sample types being investigated. Thus, e-nose devices designed specifically to detect COVID-19 disease biomarkers must contain a sensor array with sensors capable of detecting all or most of the possible VOC biomarkers present in exhaled human breath, particularly those VOCs that are most represented and present in infected individuals [163]. Consequently, a considerable amount of metabolomic research has been required to identify the VOC biomarkers most associated with COVID-19 before sensor selection in the design phase of an effective COVID-19 e-nose instrument. For example, the development of an e-nose capable of strongly detecting and differentiating various types of aldehydes as key VOC components, consistently present in breath samples from COVID-19-positive patients, would facilitate better discriminations from COVID-19-negative patients. Most e-nose devices tested for COVID-19 detection were not specifically designed and developed based on sensor array-selective sensitivities to COVID-19 identified VOCs but were e-noses developed for other applications or general uses with broad applications. This probably resulted from the need to test e-nose devices before research had been done to identify COVID-19-specific quantitative VOC biomarkers. Nevertheless, the development of a novel COVID-19 e-nose device based on specific VOC biomarkers would likely perform with much greater discrimination and accuracy than general-use e-nose devices developed to detect other diseases. Such a COVID-19-specific e-nose could potentially utilize a smaller number of highly selective sensors to specifically detect primarily COVID-19 VOC biomarkers.

The discrimination power of an e-nose device increases as the number of sensors sensitive to known target VOCs (being detected) increases. Thus, an e-nose device with 30 sensors provides greater discrimination power to distinguish sample types than one with only 10 sensors, providing that all sensors in the sensor array were specifically selected for their sensitivities to VOC metabolites targeted as known disease biomarkers for COVID-19 detection. However, the cost of e-nose devices generally increases as the number of sensors included in the sensor array increases. Consequently, as optimum e-nose configuration must be determined to balance sensor costs with desired level of discrimination power. This process can benefit from knowing which sensors provide the best discrimination power for detection of known targeted VOCs.

Most research using e-nose devices for diagnostic detection does not fully utilize smellprint patterns to their full potential as effective means of discriminating between sample types, particularly when the sample types have similar VOC composition. In these cases, discriminations of sample types require the use of various statistical analysis models and algorithms such as principal component analysis (PCA), linear discriminant analysis (LDA), and quadratic discriminant analysis (QDA), often in combination with receiver operating characteristic (ROC) curves, to evaluate the effectiveness of sample discriminations. Aroma plot PCA maps derived from e-nose output data may be used to visualize relationships between sample types through cluster analysis and distributions of data points. These plots show differences in VOC composition and chemical relatedness between different sample sources. However, comparing smellprint signatures themselves between sample types can be quite useful if the sensor array is adequate to indicate differences in the types and concentrations of VOCs between sample types. Proper sensor selection to target known VOCs and increasing the number of sensors improves sample discriminations using smellprint signatures. Smellprint signatures indicative of unique VOC profiles may be stored in known database libraries for comparisons against unknown samples for diagnostic analyses.

One of the big advantages of e-nose devices is that these instruments effectively measure differences in the molar ratios (or relative quantities) of VOCs present within air samples even when the same identical components are present in the air sample analyte [164]. This advantage is particularly useful for the detection of metabolomic (quantitative) biomarkers in which the SARS-CoV-2 virus causes an upregulation or downregulation of a specific metabolite, derived from a certain metabolic pathway, resulting in an increase or decrease in concentration of that specific metabolite within the breath specimen of COVID-19 patients compared with healthy or COVID-19-negative individuals. Of course, an e-nose, if properly designed, can detect volatile qualitative biomarkers that may be present in the breath specimen. Including sensors in the array that are preferentially sensitive to qualitative VOC biomarkers would markedly improve the sensitivity and discrimination power of the instrument to detect COVID-19 based on breath samples.

## 6. Metabolomic Research of Nonvolatile Biomarkers

Most nonvolatile COVID-19 disease biomarkers are divided into categories based on the specific organ systems where they are produced. Samprathi et al. [45] discussed different types of primarily nonvolatile COVID-19 biomarkers in terms of their pathophysiological basis and categorized them into hematological, inflammatory, coagulation, cardiac, hepatic, muscular, renal, electrolytes, biochemical, and miscellaneous, based on the specific organ systems involved. Nonvolatile COVID-19 biomarkers, such as volatile biomarkers, also may be categorized based on the particular and specific metabolic pathways or chemical processes affected by COVID-19 pathogenesis. Nonvolatile biomarkers are of sufficiently high molecular weight such that they do not dissolve as well as gases in the blood and therefore cannot be volatilize out of blood capillaries in the lungs as VOC gases that may be carried within expelled human breath. Consequently, nonvolatile biomarkers must be collected as biological fluids derived from other sources such as from the blood (serum and plasma), urine, lymph nodes, and saliva.

Considerable active research is ongoing to identify nonvolatile COVID-19 biomarkers that may be used to help improve early detection of the disease from biological samples other than from exhaled breath, particularly using blood serum and plasma specimens. Several metabolic pathways related to energy production, amino acid, and lipid metabolism have been found in several independent studies to be affected in severe COVID-19 disease cases, particularly tryptophan metabolism via the kynurenine pathway that was persistently dysregulated, suggesting the roles of several metabolites including tryptophan, kynurenine, and 3-hydroxykynurenine as potential prognostic biomarkers of the disease [22].

The most frequent and common effect of COVID-19 on human metabolic pathways is on certain types of amino acid metabolism. COVID-19’s effects on tryptophan metabolism via the Kynurenine pathway (KP) have been consistently observed in several recent studies [22]. In mammalian cells, the Kynurenine pathway controls the catabolism of tryptophan not used in protein synthesis and KP is connected to the immune system, neurological disorders, and in the diversion of tryptophan from the serotonin/melatonin pathway towards biosynthesis of the high-energy carrier, nicotinamide adenine dinucleotide (NAD) [165]. The KP enzyme, indoleamine 2,3-dioxygenase (IDO), is activated by cytokines via immune responses to SARS-CoV-2 infections that exert anti-inflammatory effects, linking KP with the immune system [166,167]. This is consistent with new findings associating KP with COVID-19 disease severity. The activity of IDO was inversely related to IL-6 levels in COVID-19 patients, but the acylcarnitines, kynurenine, and methionine sulfoxide metabolites were positively correlated with IL-6 levels [168]. Xiao et al. [169] also found increases in IL-6 and IL-10 levels associated with the nicotinate and nicotinamide metabolism in COVID-19 patients. Altered levels of kynurenine and tryptophan metabolites from tryptophan and ceramide metabolic pathways were associated with COVID-19 severity in several independent studies [165]. Increases in levels of KP metabolites, including anthranillic acid and 3-hydroxykynurenine associated with high IL-10/8 and immunosuppression in COVID-19 patients, further support the possible role of KP metabolites as potential prognostic biomarkers of COVID-19 [170]. Furthermore, Blasco et al. [171] indicated that specific metabolome profiling of COVID-19 patients supports the key role of the tryptophan and nicotinamide pathways as well as cytosine metabolism in which cytosine, described as a coordinator of cell metabolism in infections, could be useful as a highly reliable predictor for early diagnosis and a main discriminant metabolite distinguishing the metabolome profiles of COVID-19-positive vs. COVID-19-negative individuals.

Effects of SARS-CoV-2 infections on other metabolic pathways were also correlated with COVID-19 disease severity by the appearance or increase in specific associated metabolites. Shi et al. [172] investigated fatty acid metabolism and found that the levels of five fatty acids, including 2-hydroxy-3-methylbutyric acid, 3-hydroxybutyric acid, succinic acid, oleic acid, and palmitelaidic acid increased proportionally to disease severity from mild to severe cases. Barberis et al. [173] reported similar results with the appearance or proportional increases in arachidonic acid and oleic acid levels in gluconeogenesis and porphyrin metabolic pathways as disease severity increased. Levels of certain low-, high-, and very-high-density lipids (including LDLs-1, 4, 5; HDLs-1, 4; VLDL-5; and triglycerides) within the lipoprotein metabolic pathway also increased with disease severity in SARS-CoV-2-infected individuals [174]. Finally, Chen et al. [175] examined metabolites in nucleic acid and amino acid metabolism and found that the appearance or increase of certain metabolites, such as taurochenodeoxycholic acid 3-sulfate, glucoronate, and N,N,N-trimethyl–alanylproline betaine TMAP, was also correlated with COVID-19 disease severity.

Further research should help to explain why COVID-19-induced upregulation and downregulation of certain metabolic pathways causes specific nonvolatile metabolites to be significantly affected by COVID-19 pathogenesis over time following SARS-CoV-2 initial infections. Standardization of potential metabolomics-based prognostic testing methods and large-scale clinical validation are needed before these serum- and plasma-based tests can be applied in clinical settings with varying degrees of disease severity; however, metabolomics will likely play an important future role in predicting the outcome of the disease with greater certainty [22].

## 7. E-Nose COVID-19 Disease Monitoring and Recovery

The rapid physiological and health state information available from e-nose devices could help provide the potential for daily monitoring of a patient’s current condition, disease progress, response to treatments, treatment effectiveness, and rate of recovery from COVID-19. This additional e-nose information adds to the arsenal of data derived from exhaled breath VOC emissions that provide a clearer picture of physiological and metabolic activities occurring in real time and help identify which metabolic pathways and organ systems are being most impacted and affected by the disease. The science of improving metabolomic information in terms of the correlating effects of viral pathogenesis on specific metabolic pathways and the corresponding resulting changes in breath VOC composition that specifically indicate these metabolic effects will allow continuous e-nose-based disease monitoring to be developed to a higher state of utility and effectiveness. These advancements will require the acceptance and standardization of e-nose devices and procedures being used in clinical practice, the development and availability of e-nose reference databases, or libraries for specific e-nose devices that provide smellprint signatures and VOC profiles diagnostic for specific physiological conditions and disease states. These developed databases must necessarily include data for different stages of a disease on time scales that could be mathematically modeled to provide continuous data for each specific disease. Of course, many variables must be included to account for differences in host metabolic and preexisting factors. This information would add to data necessary for accurate disease detection and diagnoses by providing additional information about affected organ and metabolic systems to improve the success of targeted treatments. Thus, e-nose devices could be used not merely for disease diagnostics, but also to acquire more comprehensive information about a patient’s overall health and physiological states (throughout the body), as has been demonstrated previously for White-nose Syndrome [162].

The process of diurnal and periodic e-nose monitoring of patients’ physiological and disease states in the long-term is useful to assess progress in recovery, response to treatments, and changes in specific metabolic activities associated with specific organs to determine levels of organ dysfunctions and long COVID-19 effects. Repeated measures of physiological states require quality control procedures to assure that e-nose instrument monitoring is accurate and consistent over diverse time scales. One of the biggest problems encountered in long-term e-nose monitoring of VOC profiles is sensor drift and instability. Sensor drift has been dealt with using various strategies. Bosch et al. [176] determined that sensor drift of the C-320 e-nose could be effectively controlled by using regression models to improve accuracy in differentiating between IBD patients and controls based on corrections of six sensors in the 32-sensor array. The residuals of the regression models were applied to original patient data to produce date-corrected measurements used for subsequent data analyses. Differences in sensor outcomes between sample groups were then calculated and logistic regression analyses were performed on the sensors, which differed significantly between treatment groups. Miller et al. [3] found that using univariate and multivariate analysis on sensor response values increased the quality of sensor output responses to compensate for sensor drift. Univariate analysis allowed the capabilities of applying correction to one variable factor that caused baseline variations to avoid adverse sensor drift effects. Kwiatkowski et al. [152] recommended that periodic cleaning of gas sensor and/or treatments with UV-irradiation, pulse heating, or doping sensor sensing-layer components with metal nanoparticles (tungsten, platinum, gold, nickel, or iron) could help correct and prevent diurnal sensor drift in e-nose devices containing MOS sensor arrays [177,178].

## 8. Nonclinical Applications for COVID-19 Detections

The development of prescreening methodologies to facilitate the suppression of COVID-19 through targeted epidemiological approaches involving pre-detection prior to POCT and clinical testing is necessary to identify sources of infected individuals at ports of entry into the country, particularly travels from other highly hazardous countries considered to be major locations with active disease centers with a high risk for possibly introducing new strains of the virus. A variety of prescreening methods are possible to help facilitate detection and identification of SARS-CoV-2-infected individuals in public, POCT, and preclinical settings.

### 8.1. Canine-Assisted Disease Detection

The versatile and effective uses of canine surveillance teams for regulatory drug enforcement, law enforcement (e.g., tracking of suspects and perpetrators in crime investigations), various forensic applications, bomb detection, and for human recovery in rubble following natural disasters is well documented [163]. These live-animal, bio-detection applications of canine olfactory systems (of certain dog breeds) take advantage of the keen olfactory sensitivity and strong discriminatory capabilities of well-trained dogs. The strong potential uses of canine surveillance capabilities for coronavirus (COVID-19) early disease detection could be an effective extension of already existing, well-proven canine surveillance federal programs already developed for drug and bomb detection in airports and other international transportation depots worldwide. Additionally, considerable research has demonstrated the capabilities of canines to detect COVID-19 and a variety of other human diseases. Disease surveillance using canines in public locations would be considerably more feasible and appropriate than applications within clean medical facilities and clinical settings where sanitation is essential to minimize the occurrence of secondary infections and to prevent nosocomial (hospital-acquired) infections in potentially susceptible patients that are often in a weakened state and may be recovering from other diseases [42,43].

Studies to investigate the potential for COVID-19 canine early surveillance have been conducted by several researchers at the University of Florida [179]. Significant recent evidence suggests that canines with their highly sensitive and discriminating noses can detect COVID-19 to help confirm diagnoses. Trained dogs were competent in reproducibly recognizing SARS-CoV-2 viral infections from volatiles derived from saliva/tracheal, sweat, and urine samples [180,181,182]. Canines for the detection of COVID-19 are probably most useful in outdoor settings and in transportation depots to facilitate detections where large numbers of individuals are gathered, rather than in clinical settings where sanitation is an issue.

An official epidemiological government program for disease prevention, utilizing canine early disease surveillance of COVID-19 by federal officials at the CDC or other government agencies, could become an effective first-tier, early detection component in a more comprehensive program to detect the disease in presymptomatic humans to facilitate more effective discovery and notification of early infected, symptomless individuals that would benefit most from early treatments to help prevent the occurrence of multiple subsequent cycles of asymptomatic transmission to others before symptoms first appear. Canine surveillance could be employed in numerous public locations to help improve early disease detection, resulting in more effective COVID-19 disease management. Some locations targeted for early surveillance (with different levels of priority) could be nursing homes, entrances to medical facilities, sporting events, local and international airports, other transportation depots (buses, trains, subways, taxi, and shuttle stations), cruise lines, auditoriums, universities, and schools. The limited manpower and funding available for canine early surveillance programs would likely require implementation priorities based on the identification of locations where early disease detections would have the largest impact in mitigating epidemiological and transmission parameters considered most important in disease management.

### 8.2. User COVID-19 Self-Test Kits and Wearable E-Nose Disease Monitors

The ready availability of COVID-19 self-test kits from various sources has made it easy for individuals to obtain preliminarily results or be tentatively tested without having to visit less convenient testing facilities. Most individuals usually do not choose to utilize COVID-19 self-test kits unless they feel they have been exposed to infected individuals, have predisposing factors, or they are beginning to have virus-like symptoms and want to determine if these symptoms are likely due to a cold, flu, or COVID-19. These convenient self-test kits are quite useful to help subjects to determine if they should consult a doctor or receive early treatment, particularly if they are at higher risk due to age, race, or other predisposing factors that warrant quick action in confirming accurate diagnosis.

Much smaller specialized wearable devices such as wristwatches and smartphones with miniature e-nose devices are likely to become available in the future, providing the wearer with early warnings of possible disease. Prototypes of these devices are currently under development. Lorwongtragool et al. [183] constructed a carbon nanotube (CNTs) polymer sensor array based on inkjet printing technology. With this technology, a composite and film of CNTs/polymers were prepared as sensing layers for a chemical sensor array. Wearable e-nose devices may be used to detect VOCs and odors released from the human body to obtain physiological data and for real-time tracking of biophysical characteristics such as behavior, emotional state, and health status.

Li et al. [184] developed a smartphone-based colorimetric sensor array-type e-nose that reacts to different VOCs by a color change to create a color output pattern of different intensities. This handheld device integrates a disposable colorimetric 10-sensor array consisting of plasmonic nanocolorants and chemo-responsive organic dyes to detect key volatiles for diagnosis of diseases at the ppm level within 1 min of reaction to different chemical classes of VOCs. The camera takes a photograph of the colorimetric sensor array after the reaction to VOCs has occurred to create a permanent record of the sensor response pattern and intensity. The sensor array is then replaced for the next sample to be analyzed.

### 8.3. Standardization of COVID-19 Breath VOC Biomarker Databases

Human breathomics has emerged as an important new branch of metabolomics primarily because of greater understanding of the VOC composition in exhaled breath of healthy subjects compared with those with various disease states. Exhaled air samples are considered among the most convenient and useful specimens for the diagnosis of respiratory diseases, including viral infections, because of the ease of non-invasive collection without significant discomfort to patients [185]. A reference database, containing comprehensive human breathomics data on 913 VOCs from 2766 published reference sources, has been developed in association with several respiratory diseases such as asthma, chronic obstructive pulmonary disease (COPD), and cystic fibrosis [186]. The development of a COVID-19-specific database of VOC biomarkers should be readily possible once sufficient breath analysis data have been confirmed and replicated from many independent laboratories using identical or very similar metabolomic analytical equipment. The analytical instruments primarily used for determinations of quantitative (metabolomic) COVID-19 VOC breath biomarkers include mass spectrometry (MS) coupled to various types of pre-analysis chromatographic-type technological methods such as Proton-transfer reaction (PTR), Time-of-Flight (TOF), Thermal Desorption (TD), and Instrumental Mass Fractionation (IMF) to separate individual VOC gas analytes temporally for sequential detection and analysis. In addition, nuclear magnetic resonance (NMR) [187] and infrared (IR) spectroscopy [188] are sometimes added to help determine the molecular structure and identity of individual VOCs, particularly when multiple isomers are possible due to chiral centers.

### 8.4. Electronic-Nose Prescreening via Random Prophylactic Tests and Targeted Surveillance

Some electronic-nose devices, currently under development, will eventually become accepted and standardized for clinical practice as effective, inexpensive and easy to use breath-analysis diagnostic instruments and methods for routine uses in prophylactic screenings for COVID-19 and many other diseases. E-nose prescreening for disease may eventually become one of the first procedures performed when a patient comes into a clinic for routine checkups and for prophylactic tests such as mammograms for breast cancer, colonoscopy for colon cancer, PAP test for certain types of human papillomavirus (HPV), prostate-specific antigen (PSA) test, and other routine prophylactic tests. Simple single-sensor e-nose-like devices capable of detecting nitric oxide (NO) in a patient’s breath are already routinely used for the diagnosis of asthma in many medical clinics [137]. The versatility of breath analysis in detecting numerous diseases throughout the body make e-nose devices a simple way to obtain cheap and accurate first-diagnostic results to help prescreen and determine the need for further confirmation of diagnosis using other tests. Such electronic nose-based methods would require sufficient disease-specific reference databases on VOC smellprint libraries for all known diseases included to be screened on a routine basis. The development of e-nose databases for specific diseases could be made readily available universally to medical clinics worldwide, based on standardized methods and specific e-nose instruments used, which would greatly facilitate this reality on a global scale.

## 9. Approved Electronic Devices for COVID-19 Diagnostics in Clinical Practice

There is currently only one electronic device that has been officially approved to diagnose COVID-19 in the United States. The U.S. Food and Drug Administration (FDA) issued an emergency use authorization (EUA) on 14 April 2022 for the first COVID-19 diagnostic test that detects VOCs in human breath samples from patients with SARS-CoV-2 infections [189,190]. This FDA approval is significant because it validated the use of breath VOC analysis as an alternative test for COVID-19 detection. The test may be performed where the patient’s breath specimen is both collected and analyzed, such as in mobile testing sites, hospitals, health clinics, and doctor’s offices. Qualified and trained operators of the electronic device may conduct the test under the supervision of a licensed health care provider authorized by state law to prescribe tests. The test provides results in less than three minutes.

The instrument used to conduct this FDA-approved test, known as the InspectIR COVID-19 Breathalyzer, is about the size of a single piece of carry-on airplane luggage, and consists of a portable gas chromatography–mass spectrometry (GC-MS) device that rapidly detects the presence of the five main VOCs in exhaled breath that are known chemical biomarkers of SARS-CoV-2 infections. The identity of the five COVID-19 biomarker VOCs, targeted for detection by the instrument, was not disclosed to protect the proprietary rights of the inventor. Presumptive or unconfirmed positive test results must be confirmed with a molecular test. Negative results are evaluated in the context of a patient’s recent exposures, history, and presence of COVID-19 clinical signs and symptoms. This test should not be used as the sole basis for treatment or patient management decisions, including infection control decisions.

The performance of the InspectIR COVID-19 Breathalyzer instrument was validated in a study of 2409 individuals, including those with and without COVID-19 symptoms. The test results showed 91.2% sensitivity (the percent of positive samples the test correctly identified) and 99.3% specificity (the percent of negative samples the test correctly identified). In areas of low COVID-19 disease prevalence such as in areas where only 4.2% of individuals have proven positive for the virus, the study test had a negative predictive value of 99.6%, indicating that people who receive a negative test result are likely to be truly negative. The instrument tests performed with similar sensitivity in a follow-up clinical study focused on the Omicron variant.

The developer of the instrument, InspectIR Systems, located in Frisco, Texas, expects to produce about 100 instruments per week, each of which may evaluate up to 160 breath samples per day according to the FDA. At this level of instrument production, the COVID-19 testing capacity, using the InspectIR COVID-19 Breathalyzer alone, is expected to increase by approximately 64,000 samples per month.

There are only a few other known examples of official government-approved electronic instruments used to analyze exhaled breath for detection of COVID-19 infections in other countries of the world. The Dutch-made SpiroNose became the first breathalyzer in the world to be approved to test for COVID-19 in February 2021. This instrument is an electronic nose consisting of seven MOS sensors which measures all complex exhaled breath air specimens containing VOC components that are subsequently combined into a composite biomarker with a ROC-AUC accuracy of 0.947. Dutch health authorities determined that the SpiroNose is reliable for negative test results, but positive tests must be followed by regular PCR tests to confirm SARS-CoV-2 infections, which is the case for most positive breathalyzer test results. A mass spectrometry device known as Breathonix, like such as the InspectIR COVID-19 Breathalyzer in the U.S., was developed by a spin-off company from the National University of Singapore (NUS). It is the first instrument that has secured provisional authorization for in COVID-19 detection in Singapore. The 60 s breath test developed for this system achieved more than 90% accuracy in a Singapore-based pilot clinical trial. This mass-spec machine has a high price tag and is too big to be used in many POCT settings, but the cost was justified by the very high volume of people being COVID-19 tested as they pass to and from the adjacent country, Malaysia, through the Singapore Tuas Checkpoint Facility border crossing. An analysis of the SpiroNose performance was subsequently reported in a series of studies by deVries et al. [191].

Many other unofficial COVID-19 exhaled breath-based detection devices are under development as well, including various breathalyzer-type devices. A coronavirus breathalyzer has been defined as a diagnostic medical device enabling a user to detect the presence of SARS-CoV-2 in exhaled breath with 90% or greater accuracy [192]. The concomitant development of coronavirus breathalyzer-type devices has been ongoing since early 2020 by unrelated research groups in many countries [193,194].

## 10. Conclusions

Significant drawbacks of rRT-qPCR, the current laboratory gold standard for COVID-19 diagnosis, limit its usage and utility to application settings providing slower or delayed results. Despite its relatively high reliability, this real-time quantitative PCR method may occasionally have a false detection rate up to 25% and a false positive rate of 2.3–6.9% [195]. These limitations include its semi-invasive approach and requirements for expensive special equipment and well-trained staff as well as delayed delivery of results. The need for diagnostic methods providing quicker results has demanded the development of cheaper, more versatile, and simpler noninvasive detection tests providing high accuracy in real time for high-throughput screening (HTS). The recent GC-MS InspectIR COVID-19 Breathalyzer technology approved by the FDA for COVID-19 detection provides rapid results, but it is very expensive, effectively limiting the number of units that may be purchased and deployed. The InspectIR system does provide quicker results than PCR but does not yield more accurate results than PCR. Recent testing of artificial e-nose and animal-based olfactory methods for early detection of COVID-19 infections in humans have been investigated as possible alternatives to fill part of this technological need. Studies [196,197,198,199] evaluating applications of trained dogs have been initiated to examine canine prescreening surveillance and monitoring of COVID-19 infections of humans in public places such as in airport terminals, shopping malls, people waiting in lines assembled to receive services (including COVID-19 testing stations), at sporting events, and other outdoor situations where individuals may be approached and quickly sniffed by trained canines (similar to drug surveillance) as a prescreening first line of defense. In these cases, dogs may be trained to detect differences in the smells of healthy vs. virus-infected individuals. Such individuals testing positive for SARS-CoV-2 infections may be given notifications on the spot by trained professionals (handling surveillance dogs) of their likely infection status and be requested to report to healthcare medical services or labs for infection confirmation. Alternatively, individuals could make use of available self-test kits. In a similar way, portable e-nose units potentially may be used directly in POCT situations by COVID-19 healthcare surveillance patrol personnel to detect infections in presymptomatic individuals. This would provide a means for early detections of the disease to help mitigate transmission of the virus, particularly in asymptomatic individuals, through timely notification of infection status of individuals in public places.

Critical evaluations of COVID-19-specific epidemiological findings have suggested that the ability of the SARS-CoV-2 virus to rapidly spread from individuals who are presymptomatic or asymptomatic and indicate that effective diagnoses and isolation of individuals based on symptoms alone are ineffective in preventing ongoing spread [200,201]. Consequently, the use of more frequent population screening tests to identify infectious presymptomatic or asymptomatic individuals is a more effective strategy to break viral transmission chains to suppress the growth dynamics of the COVID-19 pandemic. Larremore et al. [202] modeled the effectiveness of repeated population COVID-19 screenings in relation to patterns of viral load kinetics by considering test sensitivities, frequency, and sample-to-answer reporting time. They demonstrated that the effectiveness of patient screening depends more on the frequency of testing and speed of reporting and that screening effectiveness is only marginally improved by high test sensitivity. They concluded that population screenings should prioritize accessibility, frequency, and rapid reporting time with only secondary emphasis on the analytical limits of COVID-19 detection. The use of simpler and cheaper e-nose devices to achieve more frequent real-time population screenings could be quite useful in supporting this improved COVID-19 disease suppression strategy.

Other types of hybrid e-nose devices, not yet investigated for COVID-19 detection, also have the potential for providing effective VOC detection at lower temperatures than conventional MOS based e-noses. Conti et al. [203] developed TiO2 nanostructure sensors combined with different polymers including poly(3,4-ethylenedioxythiophene), polystyrene sulfonate, polypyrrole, and polystyrene sulfonate to produce nanocomposites casted with gold interdigitated electrodes. Shooshtari and Salehi [204] developed carbon nanotube–titanium dioxide hybrid nanowire sensors with improved sensitivity to identify VOC gases at room temperature.

We herein summarized documented evidence and efficacy data to support a strong theoretical basis and logistical pathway by which electronic-nose and closely related VOC-detection technologies could be used for early, noninvasive detection of COVID-19 in pre-symptomatic human patients and in symptomless carriers of the disease who do not show any physical manifestations of the illness following viral infection. The many benefits of early COVID-19 detections to improve the efficiency of precision medicine, particularly using noninvasive methods, include reductions in transmission episodes, more effective regional epidemiological controls, increased options for more effective treatments, improved prognoses, and possibly reduced COVID-19-related mortality rates in all age classes, races, and ethnic groups [205]. Applications of e-nose devices for COVID-19 early detections are already possible with high accuracy and the additional use of VOC breath biomarkers to help further confirm and improve the confidence in e-nose based diagnoses.

Researchers involved in COVID-19 diagnostics continue to examine and search for VOC target metabolites in exhaled air which are characteristic of the disease to improve the development of more effective electronic sensors and chemical detectors. Metabolomics is an evolving area of science that continues to change and improve as new technologies, analytical instruments, and associated methods are developed. Application of metabolomics in investigations of infectious disease diagnostics has been promoted by the urgency of the COVID-19 pandemic. Metabolomics approaches that rely on analysis of exhaled breath VOCs from COVID-19 patients hold promise for use in large-scale screening of human populations in point-of-care (POC) settings [22]. Additional future metabolomic research will hopefully provide more mechanistic details to explain why COVID-19-induced dysregulation of certain metabolites occurs within specific metabolic pathways. Determining which VOCs are ultimately most reliable as consistent chemical biomarkers of COVID-19 infections for patients with variable personal histories, health conditions, immune systems, and nutritional states would be most useful for improving COVID-19 diagnoses using e-nose devices.

## Figures and Tables

**Table 1:** Molecular characteristics, classifications, and physiological impacts to human organs and organ systems caused by major viral diseases responsible for global pandemics.

Viral Disease ^1^	Pandemic Name ^2^	Years of Occurrence	Serotypes	Viral Family ^3^	Nucleic Acid Composition ^4^	Human Infections (inf.)/ Deaths (d.)	Main Disease Effects ^5^	Major Organ-Systems Affected	Reference
Influenza A	Spanish flu	1918–1920	A/H1N1	Orthomyxoviridae	(-) ssRNA	500 million inf., 17–50 million d.	Cytokine storms, acute secondary infections	Lungs, alimentary tract, nervous system	[50]
	Asian flu	1957–1958	H2N2	Orthomyxoviridae	(-) ssRNA	500 million inf., 1–4 million d.	Pneumonia, wobbly legs, achy limbs, headache, high fever	Lungs, muscular	[51]
	Hong Kong flu	1968–1969	H3N2	Orthomyxoviridae	(-) ssRNA	300 million inf., 1–4 million d.	Chills, fever, muscle aches, fatigue, headache, sore throat	Lungs, alimentary tract	[52]
	Swine flu	2009–2010	H1N1/09	Orthomyxoviridae	(-) ssRNA	0.7–1.4 billion inf., >575,000 d.	Tissue inflammation, Pneumonia, respiratory failure, worsening asthma and heart disease, fever, diarrhea, fatigue	Lungs, muscular	[53,54,55]
AIDS	HIV	1985-present	HIV-1, HIV-2	Retroviridae	(+) ssRNA	40 million inf., 35 million d.	ARS, diminished immunity, secondary infections and disease (especially TB, recurrent URTI, candidiasis, brain toxoplasmosis, KS, lymphoma), joint pain, rash, muscle pain Lymphadenopathy	Immune system, lungs, brain, skin	[56,57]
SARS	SARS	2002–2004	SARS-CoV-1	Coronaviridae	(+) ssRNA	8000 inf., 700 d.	ARDS, diarrhea, headaches, liver dysfunction, lymphocyte damage, myalgia, pneumonia,	Lungs, intestines, lymph nodes, spleen	[58,59]
MERS	Camel flu	2012–2020 (intermit.)	MERS-CoV	Coronaviridae	(+) ssRNA	3000 inf., 1000 d.	Diarrhea, pneumonia,	Lungs, intestines,	[60,61]
Coronavirus Disease of 2019 (COVID-19)	COVID-19	2019-present	SARS-CoV-2	Coronaviridae	(+) ssRNA	670 million inf., 6.7 million d.	Pulmonary inflammation, pneumonia, ARDS, heart failure, myocarditis, sepsis, septic shock, stress cardiomyopathy, kidney failure, loss of senses (taste and smell)	Lungs, heart, kidneys, liver, pancreas, bowel, central nervous system and brain	[62,63,64]

^1^Viral disease name abbreviations: AIDS = Acquired Immunodeficiency Syndrome; COVID-19 = Coronavirus disease 2019, originating SARS-CoV-2 infections occurred in Wuhan, China; MERS = Middle East Respiratory Syndrome; SARS = Severe Acute Respiratory Syndrome. ^2^ Pandemic name abbreviations: HIV = Human Immunodeficiency Viruses; SARS = Severe Acute Respiratory Syndrome. ^3^ Viral families: Coronaviridae (SARS-related species, Group IV); Orthoviridae (Alphainfluenzavirus species, Group V); Retroviridae (Lentivirus species, Groups N, O). ^4^ Nucleic acid RNA type and sense. ^5^ Main physical effects, including symptoms and other manifestations.

**Table 2:** Potential disease-specific metabolomic VOC biomarkers identified for possible early detection of COVID-19, caused by SARS-CoV-2 coronavirus.

VOC Sample Source	VOC-Collection Apparatus ^1^	Total Patients, N =	Chemical Analysis ^2^	COVID-19 Disease Biomarkers ^3^	Metabolomic ^4^	Chemical Class ^5^	Reference
Oral Breath	Polypropylene Haldane tube breath-sampler	98	GC-IMS	Acetone	Increase	Ketone	[48]
				Isoprene	Increase	Alkadiene, Hemiterpene	
				Heptanal	Increase	Aldehyde	
				Propanol	Increase	Alcohol	
				Propanal	Increase	Aldehyde	
				Butanone	Increase	Ketone	
				Ethanal	Increase	Aldehyde	
				Methanol	Decrease	Alcohol	
				Octanal	Increase	Aldehyde	
				1 unidentified	Increase	ND	
Expired air from endo-tracheal tube, (all mechanically ventilated patients)	Heated transfer line connected to end of endotracheal tube	28	PTR-MS	2,4-octadiene	Increase	Alkadiene	[23]
				Methylpent-2-enal	Increase	Aldehyde	
				Nonanal	Increase	Aldehyde	
				1-chloroheptane	Increase	Alkane deriv. NE	
End-tidal breath	ALTEF gas sample bags	56	GC-IMS	Acetone	Decrease	Ketone	[130]
				Propanol	Increase	Alcohol	
				10 unidentified	ND	ND	
Direct Exhaled Breath	Tedlar bag	340	PTR-TOF-MS	Nitrogen monoxide	Increase	Nitric oxide (NO)	[131]
				Butane	Increase	Alkane	
				Acetaldehyde	Increase	Aldehyde	
				Heptanal	Increase	Aldehyde	
				Ethanol	Increase	Alcohol	
				Methanol	Increase	Alcohol	
				Propionic acid	Increase	Carboxylic acid	
Direct Exhaled Breath	2-way valve, which is connected to 3L SamplePro flexFilm bag	26	GC-TOF-MS	Octanal	Increase	Aldehyde	[132]
				Nonanal	Increase	Aldehyde	
				Heptanal	Increase	Aldehyde	
				Dodecane	Increase	Alkane	
				Tridecane	Increase	Alkane	
				2-pentyl furan	Increase	Furan deriv.	
Direct Exhaled Breath	Mouthpiece with HEPA filter connected to 3 L Tedlar bag	81	TD-GC-MS	Benzaldehyde	Increase	Aromatic aldehyde	[133]
				1-propanol	Increase	Alcohol	
				3,6 methylundecane	Increase	Alkane	
				Camphene	Increase	Bicyclic monoterpene	
				β-cubebene	Increase	tricyclic sesquiterpene	
				Iodobenzene	Increase	organoiodine benzene deriv.	
				1 unidentified	ND	ND	
Direct Exhaled Breath	1.5 L Tedlar bags	85	FTIR	Methanol	Increase	Alcohol	[134]
				Ethanol	Increase	Alcohol	
				Acetaldehyde	Increase	Aldehyde	
				Carbon dioxide	Decrease	CO_2_	

^1^ Air sample collection source apparatus: Sample collected in bags or connected directly to chemical analysis devices. ^2^ Chemical analysis device abbreviations: FTIR = Fourier Transform Infra-Red Spectrometry; GC-IMS = Gas chromatography-Ion mobility spectrometry; PTR-MS = Proton-transfer-reaction-Mass spectrometry; GC-TOF-MS = Gas Chromatography-Time-of-Flight-Mass spectrometry; TD-GC-MS = Thermal Desorption-Gas chromatography-Mass spectrometry. ^3^ Disease VOC biomarker chemical identifications list. ^4^ VOC concentration in COVID-19 vs. healthy patient air samples: Increase = higher concentrations; Decrease = lower concentrations; ^5^ ND = not determined; NE = non-endogenous.

**Table 3:** Experimental electronic-nose systems tested for COVID-19 detection with performance and efficacy results.

VOC Sample Source	VOC-Collection Apparatus ^1^	Sample Size (N =) ^2^	E-Nose Device	Sensor no./Type ^3^	Accuracy ROC AUC ^4^	Application Efficacy	Reference
Oral exhaled breath	Direct breathing through Aeonose device	219	Aeonose (portable)	8 MOS 1 CO (AS-MLC) 1 NO2 (AS-MLN) 1 VOC (AS-MLX)	0.74	Pre-operative screening of SARS-CoV-2 infections	[155]
Oral exhaled breath	Hand-held breathalyzer system	140	NaNose	8 GNP	0.76; 0.95	Differentiated COVID-19 from controls and other lung infections	[44]
Oral exhaled breath	BioVOC breath sampler	56	Experimental	3 MEMS, 1 MOS	0.79–0.89	Differentiated COVID-19 from controls	[152]
Intranasal air extraction	Nasal sampling valve	54	PEN3 eNose	10 MOS	0.58; 0.63	57% false + precludes clinical use	[156]
Oral exhaled breath	T-piece valve; 5L Nalophan^TM^ bags	55	EOS-AROMA	4 MOS	0.81	Differentiated respiratory failure vs. controls and asymptomatic SARS-CoV-2 infections	[157]
Oral exhaled breath	Mask-integrated sampling bag	615	GeNose C19 (portable)	10 MOS	0.88–0.95	Screening tool to distinguish patients with COVID-19 from other diseases	[158]
Oral exhaled breath	3 L Tedlar sampling bags	123–132 (5 trials)	Cyranose 320 (portable)	32 CBPC	1.00	Post-COVID-19 patient screening	[159]
Oral exhaled breath	1.4 L metallized plastic bag	102	Cyranose-320 (portable)	32 CBPC	0.98; 1.00	Post-coronavirus disease syndrome (PCS)	[45]
Oral exhaled breath	1 L medical-grade PVC sampling bag	460	GeNose C19 (portable)	10 MOS	0.86	COVID- 19 detection	[160]

^1^ Human breath air sampling methods. ^2^ Sample size: Total number of patients evaluated from several sampling classes including controls, SARS-CoV-2 infected, and other respiratory diseases. ^3^ Sensor type abbreviations: CBPC = carbon black polymer composite; GNP = gold nanoparticle; MEMS = microelectromechanical systems; MOS = metal oxide semiconductor. ^4^ Accuracy of the specified diagnostic application method based on Receiver Operating Characteristic (ROC) area under the curve (AUC).

## Abbreviations

List of Acronyms and Abbreviations: (alphabetical).

ARDS	Acute respiratory distress syndrome
AUC	Area under the curve
CBPC	Carbon black polymer composite
ECMO	Extracorporeal membrane oxygenation
FTIR	Fourier Transform Infra-Red Spectrometry
GC-IMS	Gas chromatography-Ion mobility spectrometry
GC-MS	Gas chromatography mass spectrometry
GCxGC-MS	Comprehensive 2-dimensional gas chromatography–mass spectrometry
GC-TOF-MS	Gas chromatography–Time-of-Flight–mass spectrometry
GNP	Gold nanoparticle
HTS	High throughput screening
IR	Infrared spectroscopy
LC-MS	Liquid chromatography–mass spectrometry
LC-MS/MS	Liquid chromatography–tandem mass spectrometry
MEMS	Microelectromechanical systems
MOS	Metal oxide semiconductor
NMR	Nuclear magnetic resonance spectroscopy
PTR-MS	Proton-transfer-reaction–Mass spectrometry
ROC	Receiver operating characteristic
rRT-qPCR	Real time reverse-transcription quantitative polymerase chain reaction
TD-GC-MS	Thermal Desorption–Gas chromatography–Mass spectrometry
UHPLC-MS	Ultra-high-performance liquid chromatography–mass spectrometry
UHPLC-MS/MS	Ultra-high performance liquid chromatography–tandem mass spectrometry
UPLC-MS/MS	Ultra-performance liquid chromatography–tandem mass spectrometer
